# New insights into radioresistance in breast cancer identify a dual function of miR‐122 as a tumor suppressor and oncomiR

**DOI:** 10.1002/1878-0261.12483

**Published:** 2019-04-18

**Authors:** Isidro X. Perez‐Añorve, Claudia H. Gonzalez‐De la Rosa, Ernesto Soto‐Reyes, Fredy O. Beltran‐Anaya, Oscar Del Moral‐Hernandez, Marisol Salgado‐Albarran, Oscar Angeles‐Zaragoza, Juan A. Gonzalez‐Barrios, Daniel A. Landero‐Huerta, Margarita Chavez‐Saldaña, Alejandro Garcia‐Carranca, Nicolas Villegas‐Sepulveda, Elena Arechaga‐Ocampo

**Affiliations:** ^1^ Posgrado en Ciencias Naturales e Ingenieria Division de Ciencias Naturales e Ingenieria Universidad Autonoma Metropolitana Mexico City Mexico; ^2^ Departamento de Ciencias Naturales Universidad Autonoma Metropolitana, Unidad Cuajimalpa Mexico City Mexico; ^3^ Laboratorio de Genomica del Cancer Instituto Nacional de Medicina Genomica Mexico City Mexico; ^4^ Laboratorio de Virologia y Epigenetica del Cancer Facultad de Ciencias Quimico Biologicas Universidad Autonoma de Guerrero Chilpancingo Mexico; ^5^ Unidad de Radioterapia Instituto Nacional de Cancerologia Mexico City Mexico; ^6^ Laboratorio de Medicina Genomica Hospital Regional “1° de Octubre” Mexico City Mexico; ^7^ Laboratorio de Biologia de la Reproduccion Instituto Nacional de Pediatría Mexico City Mexico; ^8^ Unidad de Investigacion Biomedica en Cancer‐Laboratorio de Virus y Cancer Instituto Nacional de Cancerologia Mexico City Mexico; ^9^ Departamento de Biomedicina Molecular Centro de Investigacion y de Estudios Avanzados (CINVESTAV) Mexico City Mexico

**Keywords:** breast cancer, microRNAs, miR‐122, radioresistance

## Abstract

Radioresistance of tumor cells gives rise to local recurrence and disease progression in many patients. MicroRNAs (miRNAs) are master regulators of gene expression that control oncogenic pathways to modulate the radiotherapy response of cells. In the present study, differential expression profiling assays identified 16 deregulated miRNAs in acquired radioresistant breast cancer cells, of which miR‐122 was observed to be up‐regulated. Functional analysis revealed that miR‐122 has a role as a tumor suppressor in parental cells by decreasing survival and promoting radiosensitivity. However, in radioresistant cells, miR‐122 functions as an oncomiR by promoting survival. The transcriptomic landscape resulting from knockdown of miR‐122 in radioresistant cells showed modulation of the *ZNF611*,* ZNF304*,* RIPK1*,* HRAS*,* DUSP8* and *TNFRSF21* genes. Moreover, miR‐122 and the set of affected genes were prognostic factors in breast cancer patients treated with radiotherapy. Our data indicate that up‐regulation of miR‐122 promotes cell survival in acquired radioresistant breast cancer and also suggest that miR‐122 differentially controls the response to radiotherapy by a dual function as a tumor suppressor an and oncomiR dependent on cell phenotype.

**Abbreviations**
GO: Gene Ontology
miRNAs: microRNAs
oncomiRs: oncogenic miRNAs
qRT‐PCR: quantitative reverse transcriptase‐polymerase chain reaction
RFS: relapse‐free survival
SF: surviving fraction
TCGA: The Cancer Genome Atlas
TLDAs: TaqMan low‐density arrays
TNBC: triple‐negative breast cancer

## 1 Introduction

Radiotherapy, in addition to surgery and chemotherapy, remains the core of the current clinical management of breast cancer. Although radiotherapy is effective in most patients, some of them will develop recurrent disease because of radioresistant tumor cells (Jameel *et al*., 2004). Radiotherapy is an extrinsic factor that affects the behavior of the breast cancer cells themselves. When cells avoid the effect cytotoxic of radiation, cell growth is induced and spreads, resulting in a progression or recurrence of tumors in patients (Moran and Haffty, 2002; Torres‐Roca *et al*., 2015). To overcome this problem, it is necessary to detemrine the mechanisms of resistance to radiotherapy. Several studies have demonstrated that tumor recurrence and progression as a consequence of radioresistance can be regulated by microRNAs (miRNAs) (Arechaga‐Ocampo *et al*., 2017; Metheetrairut and Slack, 2013). miRNAs are master regulators of gene expression; moreover, they have a role in the regulation of carcinogenesis and the control of response to chemo‐ and radiotherapy in breast cancer (Zhang *et al*., 2014). miRNAs are short, 18–25 nucleotides in length, noncoding RNA molecules that regulate gene expression by suppressing mRNA translation and reducing mRNA stability, usually via imperfect complementary base pairing to the 3′‐UTR (Bartel, 2004). miRNAs in cancer are classically categorized as either tumor suppressive or oncogenic. Generally, oncogenic miRNAs (oncomiRs) are overexpressed in tumors, whereas tumor‐suppressive miRNAs are repressed. When these tumor‐suppressor miRNAs or oncomiRs are stimulated or inhibited, respectively, cancer cell growth, proliferation, metastasis and survival may be significantly reduced via the control of pro‐oncogenic factors (Svoronos *et al*., 2016). miR‐122 is frequently down‐regulated in breast cancer and has been related to tumor suppressor activity in breast cancer. Up‐regulation of miR‐122 suppressed cell growth and cell‐cycle progression in breast cancer cell lines and suppressed tumorigenesis *in vivo* by targeting *IGF1R* and regulating the PI3K/Akt/mTOR/p70S6K pathway (Wang *et al*., 2012). However, the crucial roles and underlying the mechanisms of miR‐122 with respect to radioresistance of breast cancer remain unclear. In the present study, we report the generation of an isogenic model of acquired radioresistant human breast cancer cells, as well as functional approaches aiming to identify the molecular changes in miRNAs that may explain this phenotype. We demonstrate that miR‐122 has a dual function in breast cancer because it has tumor suppressor activity as a result of sensitizing parental cells to radiation, although it functions as an oncomiR in radioresistant breast cancer cells by promoting cell survival.

## 2 Materials and methods

### 2.1 Cell lines

Human breast cancer cell lines MCF‐7 and MDA‐MB‐231 were obtained from the ATCC (Manassas, VA, USA) (# HTB‐22 and HTB‐26). MCF‐7, MCF‐7RR, MDA‐MB‐231 and MDA‐MB‐231RR cell lines were cultured in Dulbecco's modified Eagle's medium (Gibco, Gaithersburg, MD, USA) supplemented with 10% fetal bovine serum, 100 IU·mL^−1^ penicillin and 100 μg·mL^−1^ streptomycin at 37 °C in a 5% CO_2_ atmosphere.

### 2.2 Establishment of radioresistant breast cancer cells

MCF‐7RR and MDA‐MB‐231RR cell lines were established from their parental MCF‐7 and MDA‐MB‐231 cells. 1 × 10^6^ parental cells were irradiated with a linear accelerator (Clinac 600; Varian Inc., Palo Alto, CA, USA) available at the National Institute of Cancer in Mexico City. Cells received 15 sequential fractions of 2 Gy·week^−1^, allowing irradiated cell populations a period of recovery between exposures. Non‐irradiated controls were handled identically to the irradiate cells without radiation exposure. All of the experiments were performed within 4–10 passages after the final irradiation.

### 2.3 Clonogenic survival assay

About 3 × 10^5^ cells were irradiated and, after 24 h of radiation, 1000 cells per well were seeded in six‐well tissue culture plates. The cells were cultured for 10–12 days. Colonies were fixed with 7 : 1 methanol/acetic acid, stained with 0.05% crystal violet and counted. The surviving fraction (SF) was calculated according to Franken *et al*. (2006). The SF of cells was plotted on a log scale.

### 2.4 miRNAs expression profile analysis

Expression of 667 miRNAs was analyzed by a quantitative reverse transcriptase‐polymerase chain reaction (qRT‐PCR) using the Megaplex TaqMan Low‐Density Arrays (TLDAs), version 2.0, system (Applied Biosystems, Foster City, CA, USA). Briefly, 100 ng of total RNA was retro‐transcribed using stem‐loop primers and a pre‐amplification step was added so that the minimum amounts of miRNAs were detected. qPCR assays were performed using GeneAmp System 9700 (Applied Biosystems).

### 2.5 qRT‐PCR

The expression of individual miRNAs was evaluated via qRT‐PCR using the Stem‐loop RT miRNA assay (Applied Biosystems). About 100 ng of total RNA was retro‐transcribed using the looped RT primer (Applied Biosystems) in accordance with the manufacturer's protocol. Detection for miR‐122, miR‐10a, miR‐222, miR‐222*, miR135b, miR‐135b*, miR‐196b and miR‐934 was performed using TaqMan Universal PCR Master Mix (Applied Biosystems). qPCR was carried out in 7500 Real‐Time PCR System (Applied Biosystems). The expression of miRNA was determined using the comparative *C*_t_ ($2^{-\Delta \Delta C_{t}}$) method. RNU44 was used as a control for normalization of data.

### 2.6 Transfections

MCF‐7 and MDA‐MB‐231 cells were transfected with mimic‐miR122 (Ambion, Austin, TX, USA) 10 nm, whereas MCF‐7RR and MDA‐MB‐231RR cells were transfected with antagomiR‐122 (Ambion) 30 nm. Mimic‐miR122 and antagomiR‐122 were diluted in Opti‐Mem (Invitrogen, Carlsbad, CA, USA), scramble sequence was used as a control and Lipofectamine 2000 (Invitrogen) was used as transfection agent. The expression of miR‐122 was evaluated 48 h post‐transfection by qRT‐PCR. After transfection, cells were irradiated with 4 Gy of irradiation. The subsequent clonogenic assay was performed as described previously in the section 2.3.

### 2.7 Microarray processing and data analysis

Total RNA was obtained from MCF‐7RR and MCF‐7RR cells transfected with antagomiR‐122. Equimolar concentrations of total RNA from three independent experiments were mixed and the transcriptional profiles were analyzed using the Affymetrix GeneChip Human Gene 1.0 ST array (Affmetrix, Santa Clara, CA, USA) in accordance with the manufacturer's instructions. Arrays were scanned using a Genechip Scanner 3000 7G (Affmetrix). The data were analyzed with the Robust Multichip Analysis algorithm using the default analysis settings (Affmetrix) and global scaling as the normalization method. To define the differential expression profile, transcriptome analysis console software (Affmetrix) was used. Genes with fold change > 1.3 or < −1.3 and with *P* < 0.05 by ANOVA were considered significantly altered between the conditions (MCF‐7RR and MCF‐7RR cells transfected with antagomiR‐122). Microarray raw data tables have been deposited at the National Center for Biotechnology Information Gene Expression Omnibus (https://www.ncbi.nlm.nih.gov/geo/query/acc.cgi?acc=GSE120171).

### 2.8 Bioinformatic analysis

Verified miRNAs targets were obtained by miRTarBase (http://mirtarbase.mbc.nctu.edu.tw) and predicted miRNAs targets from the miRWalk database (http://zmf.umm.uni-heidelberg.de/apps/zmf/mirwalk). Only miRNA‐target interactions identified by at least three algorithms were considered. david, version 6.7 (https://david-d.ncifcrf.gov) and panther pathway (http://www.pantherdb.org/pathway) were used to identify components of signaling pathways and Gene Ontology (GO) for biological processes or molecular functions. The analysis of biological network enrichment of the modulated genes obtained by microarrays assays was performed by cytoscape (https://cytoscape.org) using the Key Pathway Miner App (Alcaraz *et al*., 2014). In this analysis, a value of *K* = 6 was used.

### 2.9 The Cancer Genome Atlas (TCGA) data analysis

The RNA sequencing data from samples of 491 breast cancer patients were downloaded from the TCGA database (https://portal.gdc.cancer.gov). First‐line treatment and/or additional radiotherapy, tumor status and follow‐up days were considered. Total population was stratified according to low or high expression of mir‐122, *ZNF611*,* ZNF304*,* RIPK1*,* TNFRSF21*,* DUSP8* and *HRAS*. Kaplan–Meier analysis was used for relapse‐free survival (RFS) curves and log‐rank tests were employed to analyze the differences between curves. The results were confirmed by Cox proportional‐Hazard regression analyses.

### 2.10 Western blot assays

Following cell transfection, total protein was extracted, separated on SDS/PAGE and blotted onto nitrocellulose membranes (Bio‐Rad, Hercules, CA, USA). Membranes were probed with specific primary antibodies [ZNF611 (Abcam, Cambridge, MA, USA); ZNF304 (Abcam); RIPK1 (BD Transduction Laboratories, Lexington, KT, USA); DUSP8 (Santa Cruz Biotechnology, Santa Cruz, CA, USA); HRAS (Abcam); TNFR21 (Santa Cruz); and β‐actin (Cell Signaling Technology, Beverly, MA, USA)], followed by horseradish peroxidase‐conjugated secondary antibodies anti‐mouse (Zymed Laboratories Inc., San Francisco, CA, USA) or anti‐rabbit (Zymed). Immunodetection was by chemiluminescence (Super Signal^®^ West Femto; Thermo Scientific, Waltham, MA, USA). Densitometry analysis was performed using imagej, version 1.45 (National Institute of Health, Bethesda, MD, USA).

### 2.11 Statistical analysis

All results were derived from three independent experiments, which were plotted as the mean ± SD. The comparison between the groups was performed using ANOVA for all analyzes. *P* ≤ 0.05 was considered statistically significant. All statistical analyses were performed using spss, version 17.0 (SPSS Inc., Chicago, IL, USA).

## 3 Results

### 3.1 Establishment of radioresistant MCF‐7 and MDA‐MB‐231 breast cancer cells

**Figure 1:**
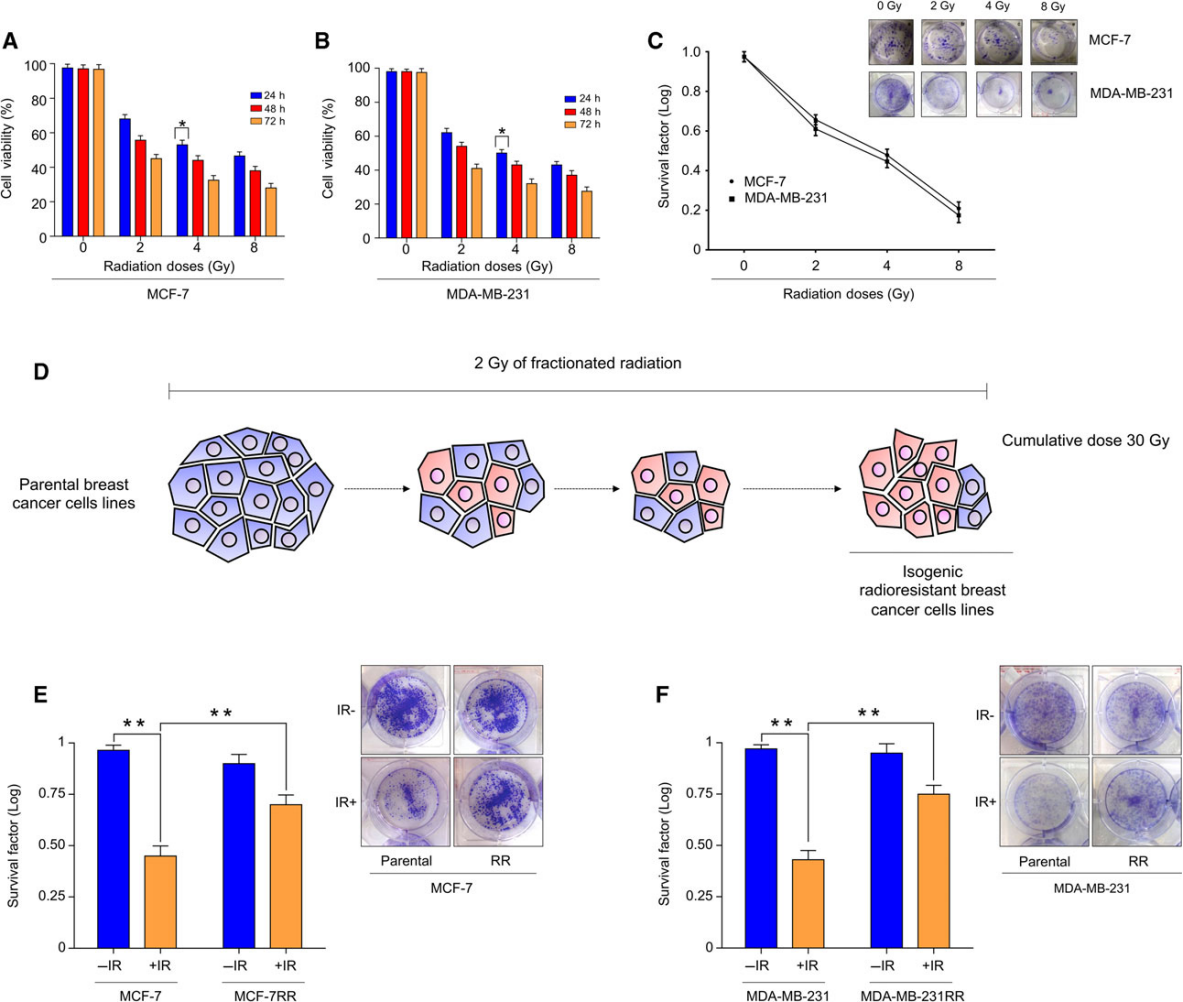
Establishment of an isogenic model of acquired radioresistant breast cancer cells. Proliferation of parental (A) MCF‐7 and (B) MDA‐MB‐231 cells was assessed by the MTT assay for 24, 48 and 72 h after irradiation with increasing doses (0, 2, 4 and 8 Gy) of irradiation. (C) Clonogenic survival of parental MCF‐7 and MDA‐MB‐231 cells was assessed by colony formation in response to treatment with increasing doses (0, 2, 4 and 8 Gy) of irradiation. (D) Schematic overview of fractionated treatment schedule for establishment of radioresistant breast cancer cells. Parental cells were exposed to 2 Gy of irradiation to reach a total dose of 30 Gy. Radioresistance of MCF‐7RR and MDA‐MB‐231RR cells was confirmed by clonogenic assays after 4 Gy of irradiation. The SF of irradiated (IR+) MCF‐7RR (E) and MDA‐MB‐231RR (F) cells was normalized by the SF of non‐irradiated (IR−) cells. Representative images of the results of the clonogenic assays for MCF‐7RR and MDA‐MB‐231RR cells are shown in (E) and (F). Error bar indicates the SD from three independent experiments. **P < 0.01; *P < 0.05 by Student's *t*‐test.

To determine a mean lethal dose of irradiation, MCF‐7 and MDA‐MB‐231 cells were evaluated by MTT [3‐(4,5‐dimethylthiazol‐2‐yl)‐2,5‐diphenyl‐tetrazolium bromide] and clonogenic assays following increased radiation dose (2–8 Gy). The results showed that cell proliferation was significantly reduced at different time points (0, 24, 48 and 72 h) and at increased radiation doses (0, 2, 4, 8 Gy) compared to control cells. Proliferation diminished to 53 ± 0.07% in MCF‐7 (Fig. 1A) and 58 ± 0.05% in MDA‐MB‐231 (Fig. 1B) cells at 4 Gy at 48 h. The results from clonogenic assays showed a significant reduction in survival (Fig. 1C) of MCF‐7 (0.48 ± 0.005) and MDA‐MB‐231 (0.44 ± 0.005) cells at 4 Gy compared to non‐irradiated cells. These results indicated that 4 Gy of radiation represents a mean lethal dose for MCF‐7 cells and MDA‐MB‐231 cells. Therefore, a radiation dose of 4 Gy was used for all subsequent experiments. To establish the isogenic model of radioresistance, MCF‐7 and MDA‐MB‐231 cells were exposed to 2 Gy‐fractionated irradiations to a cumulative dose of 30 Gy (Fig. 1D). At the end of this process, radioresistance of the resulting cell population, designated as MCF‐7RR and MDA‐MB‐231RR, was confirmed by clonogenic assays after single doses of 4 Gy of radiation. Acquired radioresistance of MCF‐7RR and MDA‐MB‐231RR cells was further indicated by an increase of survival after 4 Gy of radiation compared to parental cells. The SF of the MCF‐7RR (Fig. 1E) and MDA‐MB‐231RR (Fig. 1F) cells was 0.70 and 0.75, respectively; whereas, for parental MCF‐7 and MDA‐MB‐231 cells, the SF was 0.47 and 0.45, respectively. These data confirmed that the population resulting from the cells exposed to long‐term therapeutic fractionated irradiation developed a radioresistant phenotype.

### 3.2 miR‐122 is overexpressed in therapy‐induced radioresistant breast cancer cells

**Figure 2:**
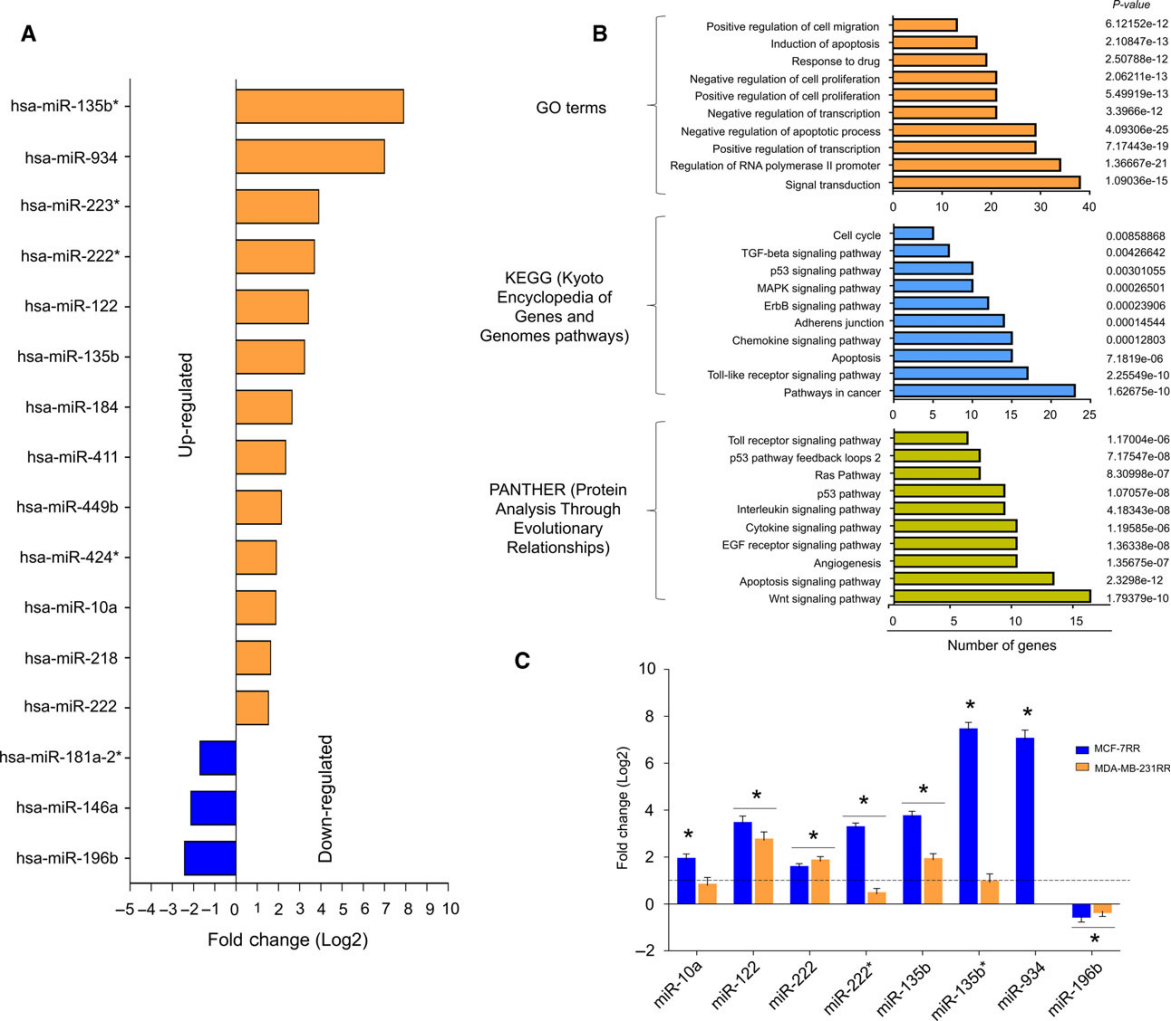
Radioresistant breast cancer cells show the differential expression profile of miRNAs. (A) miRNA expression profile in MCF‐7RR cells. (B) GO, signaling pathways and biological processes controlled by deregulated miRNAs in MCF‐7RR cells. (C) Validation of the expression of a set of miRNAs in MCF‐7RR and MDA‐MB‐231RR cells was performed by qRT‐PCR. All values were normalized using RNU44 as an internal control. The expression data were normalized using the parental MCF‐7 and MDA‐MB‐231 cells. A dotted line indicates the threshold of the normalized data. Data are presented as the mean ± SD of three independent experiments. *P < 0.01 by ANOVA.

To identify miRNAs associated with radioresistance in breast cancer, we analyzed the expression of 667 miRNAs by PCR array analysis in MCF‐7RR cells. The expression of 16 miRNAs was modulated (Fig. 2A). miR‐135b*, miR‐934, miR‐223*, miR‐222*, miR‐122, miR‐135b, miR‐184, miR‐411, miR‐449b, miR‐424*, miR‐10a, miR‐218 and miR‐222 miRNAs were significantly overexpressed (fold change ≥ 1.5), whereas miR‐181a‐2*, miR‐146a and miR‐196b were down‐regulated (fold change ≤ −1.5) (Table 1). Individual qRT‐PCR assays using RNA samples from different clones of MCF‐7RR cells further confirmed the array results (Fig. S1). Verified target genes of miRNAs were obtained from databases and previous studies (Table 2). GO and enrichment analysis indicated that the set of miRNAs could modulate biological pathways such as cell migration, signal transduction, apoptosis and survival (Fig. 2B). The expression of the most significantly modulated miRNAs identified in MCF‐7RR cells was also evaluated in MDA‐MB‐231RR cells. Remarkably, overexpression of miR‐122, miR‐222 and miR‐135b, as well as downregulation of miR‐196b, was likewise observed in MDA‐MB‐231RR cells; conversely, miR‐222* and miR‐934 were deeply suppressed in MDA‐MB‐231RR cells (Fig. 2C). These results suggested that variation of mir‐122, miR‐222, miR‐135b and miR‐196b expression might be a relevant phenomenon in the acquired radioresistance of breast cancer cells.

**Table 1:** miRNAs with modulated expression in breast cancer cells MCF‐7RR

miRNA	Fold change (log 2)	*R* (*P* value)*	Chromosome
Down‐regulated			
hsa‐miR‐196b	−2.43	0.0113	7p15.2
hsa‐miR‐146a	−2.13	0.05	5q34
hsa‐miR‐181a‐2*	−1.7	0.0091	9q33.3
Up‐regulated			
hsa‐miR‐222	1.53	0.0083	Xp11.3
hsa‐miR‐218	1.63	0.0097	4p15.31
hsa‐miR‐10a	1.88	0.032	17q21.32
hsa‐miR‐424*	1.9	0.0375	Xq26.3
hsa‐miR‐449b	2.14	0.017	5q11.2
hsa‐miR‐411	2.34	0.0452	14q32.31
hsa‐miR‐184	2.64	0.017	15q25.1
hsa‐miR‐135b	3.23	0.0068	1q32.1
hsa‐miR‐122	3.41	0.0282	18q21.31
hsa‐miR‐222*	3.7	0.0169	Xp11.3
hsa‐miR‐223*	3.9	0.0014	Xq12
hsa‐miR‐934	7	0.0529	Xq26.3
hsa‐miR‐135b*	7.9	0.015	1q32.1

*P < 0.01 by ANOVA.

**Table 2:** Verified targets genes of miRNAs modulated in MCF‐7RR cells

	Genes
Down‐regulated	
hsa‐miR‐196b	*HOXB8*,* HOXC8*,* CD8A*,* HOXA9*,* HOXA9*,* MEIS1*,* FAS*,* ETS2*,* RDX*,* HOXB7*
hsa‐miR‐146a	*CXCR4*,* TLR2*,* FADD*,* TRAF6*,* IRAK1*,* ROCK1*,* BRCA2*,* BRCA1*,* NFKB1*,* CDKN1A*,* EGFR*,* CD40LG*,* FAS*,* ERBB4*,* SMAD4*,* TLR4*,* WASF2*,* STAT1*,* UHRF1*,* L1CAM*,* SMN1*,* CARD10*,* COPS8*,* ELAVL1*,* NUMB*,* PTGS2*,* CCL5*,* PTGES2*,* CNOT6L*,* SIKE1*,* CXCL12*,* PRKCE*,* RAC1*,* LAMC2*,* COX2*,* RNF11*
hsa‐miR‐181a‐2*	Not reported
Up‐regulated	
hsa‐miR‐222	*STAT5A*,* CDKN1B*,* SOD2*,* MMP1*,* FOXO3*,* CDKN1C*,* KIT*,* PPP2R2A*,* TIMP3*,* FOS*,* ICAM1*,* ESR1*,* BBC3*,* PTEN*,* SELE*,* DIRAS3*,* ETS1*,* DICER1*,* RECK*,* TRPS1*,* CERS2*,* GJA1*,* SSX2IP*,* DKK2*,* VGLL4*
hsa‐miR‐218	*LAMB3*,* LASP1*,* IKBKB*,* SP1*,* VOPP1*,* BIRC6*,* ACTN1*,* STAM2*,* CDKN1B*,* BIRC5*,* GJA1*,* ROBO1*,* RICTOR*,* SOST*,* SFRP2*,* HOXB3*,* DKK2*,* TOB1*,* CDK6*,* BMI1*,* LEF1*,* MITF*,* PDGFRA*,* GLI2*,* OTUD7B*,* RUNX2*,* CDH2*,* EGFR*,* RET*,* SH3GL1*
hsa‐miR‐10a	*HOXA1*,* USF2*,* MAP3K7*,* BTRC*,* SRSF1*,* TRA2B*,* CHL1*,* PTEN*,* PIK3CG*
hsa‐miR‐424*	Not reported
hsa‐miR‐449b	*SIRT1*,* CCNE2*,* MET*,* GMNN*,* HDAC1*
hsa‐miR‐411	Not reported
hsa‐miR‐135b	*APC*,* KLF4*,* MAFB*,* CASR*,* PPP2R5C*,* SMAD5*,* LZTS1*,* MID1*,* MTCH2*,* ACVR1B*,* BMPR2*,* TGFBR1*
hsa‐miR‐184	*AKT2*,* INPPL1*,* NFATC2*,* SOX7*,* EIF2C2*,* MYC*,* BCL2*,* EZR*
hsa‐miR‐122	*CYP7A1*,* IGF1R*,* SRF*,* RAC1*,* RHOA*,* ANK2*,* NFATC2IP*,* ENTPD4*,* ANXA11*,* ALDOA*,* RAB6B*,* RAB11FIP1*,* FOXP1*,* MECP2*,* NCAM1*,* UBAP2*,* TBX19*,* AACS*,* DUSP2*,* ATP1A2*,* MAPK11*,* FUNDC2*,* AKT3*,* TPD52L2*,* GALNT10*,* G6PC3*,* AP3M2*,* SLC7A1*,* XPO6*,* FOXJ3*,* SLC7A11*,* TRIB1*,* EGLN3*,* NUMBL*,* ADAM17*,* DSTYK*,* FAM117B*,* BCL2L2*,* PRKAB1*,* ADAM10*,* ACVR1C*,* PRKRA*,* WNT1*,* PTPN1*,* NT5C3A*,* P4HA1*,* PKM*,* CLIC4*,* MEF2D*,* AXL*,* NOD2*,* FUT8*
hsa‐miR‐222*	*STAT5A*,* CDKN1B*,* SOD2*,* MMP1*,* FOXO3*,* CDKN1C*,* KIT*,* PPP2R2A*,* TIMP3*,* TNFSF10*,* FOS*,* ICAM1*,* ESR1*,* BBC3*,* PTEN*,* SELE*,* DIRAS3*,* ETS1*,* DICER1*,* RECK*,* TRPS1*,* CERS2*,* GJA1*,* SSX2IP*,* DKK2*,* ADAM1A*,* MGMT*,* VGLL4*
hsa‐miR‐223*	Not reported
hsa‐miR‐934	Not reported
hsa‐miR‐135b*	*APC*,* KLF4*,* MAFB*,* CASR*,* PPP2R5C*,* SMAD5*,* LZTS1*,* MID1*,* MTCH2*,* ACVR1B*,* BMPR2*,* TGFBR1*

### 3.3 miR‐122 increases radiosensitivity in parental breast cancer cells and in breast cancer patients treated with radiotherapy

**Figure 3:**
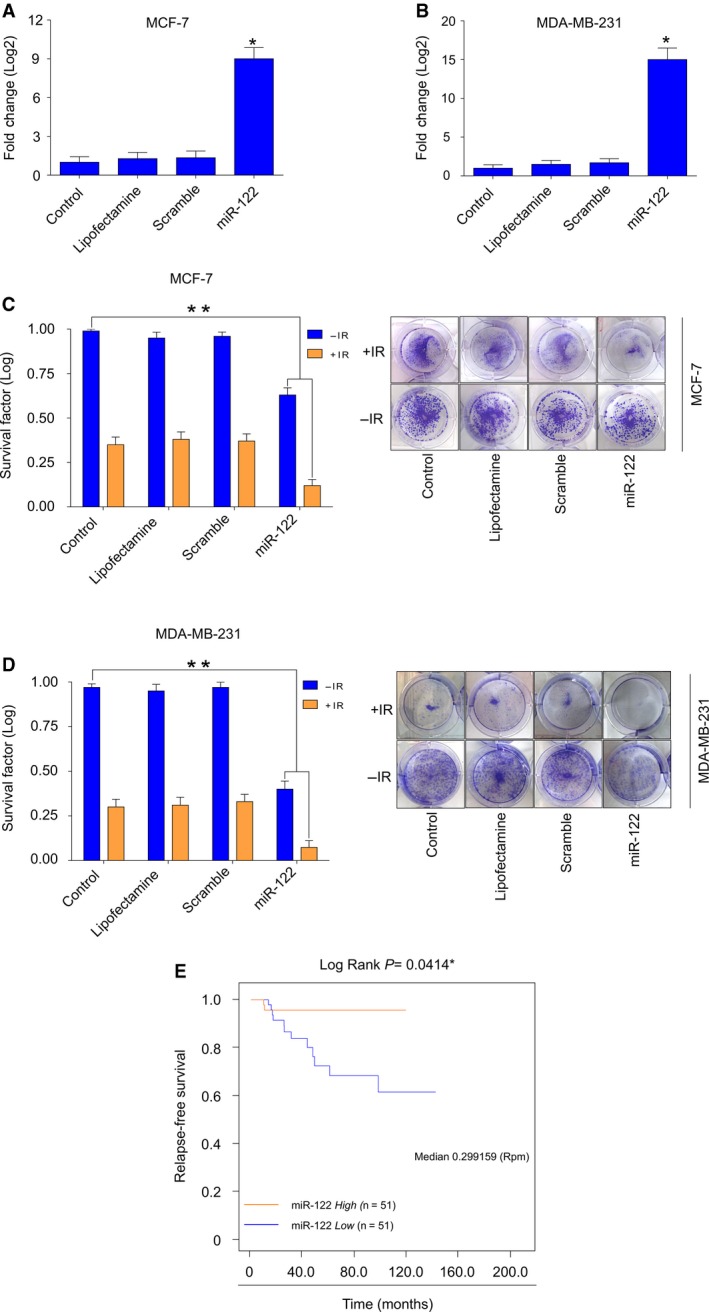
MiR‐122 promotes radiosensitivity in parental breast cancer cells. Increased expression of miR‐122 in parental (A) MCF‐7 and (B) MDA‐MB‐231 cells transfected with mimic‐miR122 was verified by qRT‐PCR assays. All values were normalized using RNU44 as an internal control. Mimic‐miR122‐transfected cells were evaluated for a radioresponse by clonogenic survival. Data for SF of transfected (C) MCF‐7 and (D) MDA‐MB‐231 cells irradiated (+IR) with 4 Gy of X‐ray are shown. Data were normalized using non‐irradiated cells (−IR). Representative images of the clonogenic assays results of MCF‐7 and MDA‐MB‐231 cells are shown in (C) and (D). Data are presented as the mean ± SD of three independent experiments. *P < 0.05; **P < 0.01 by ANOVA. (E) Kaplan–Meier analysis of the breast cancer patients with tumors positive or negative for miR‐122 expression who received radiotherapy treatment. Curves were compared using a log‐rank test *P < 0.01. Rpm, reads per million.

miR‐122 has been described as a tumor suppressor and its downregulation is a common event in breast cancer (Wang *et al*., 2012). Conversely, in the present study, overexpression of miR‐122 was observed in both radioresistant breast cancer cells. To investigate whether miR‐122 might increase the radioresistance of breast cancer cells, we performed assays of gain‐of‐function in parental MCF‐7 and MDA‐MB‐231 cells. We were able to overexpress miR‐122 in parental MCF‐7 (Fig. 3A) and MDA‐MB‐231 cells (Fig. 3B) using a mimic‐miR122. Next, we evaluated the survival potential of these cells in response to radiotherapy by clonogenic assays. The results showed that, in non‐irradiated cells, the overexpression of miR‐122 alone but not scrambled transfected or control untransfected significantly decreased the survival MCF‐7 (SF = 0.63) (Fig. 3C) and MDA‐MB‐231 (SF = 0.4) (Fig. 3D) cells. Remarkably, cells transfected with mimic‐miR122 treated with radiotherapy significantly diminished their survival potential (MCF‐7: SF = 0.12; MDA‐MB‐231: SF = 0.07) (Fig. 3C,D). These results revealed that miR‐122 is capable of sensitizing the breast cancer cells to radiotherapy. To investigate the clinical relevance of the miR‐122 expression levels, we performed Kaplan–Meier analysis for RFS of breast cancer patients treated with radiotherapy. Data for 102 patients who received radiotherapy as a first‐line treatment obtained from the TCGA database were randomly categorized into two groups according to positive or null expression of miR‐122. The results revealed that patients with a positive expression of miR‐122 and who had received radiotherapy had a significantly better RFS than those with negative expression of miR‐122 (Fig. 3E), suggesting that patients with null expression of miR‐122 were significantly associated with poor prognoses after radiotherapy. These results were in agreement with our findings obtained *in vitro*, in which miR‐122 sensitized breast cancer cells to irradiation. Furthermore, the results obtained *in vivo* suggested that the expression of miR‐122 might be predictor biomarker for RFS in breast cancer patients treated with radiotherapy.

### 3.4 miR‐122 knockdown overcomes acquired radioresistance in breast cancer cells

**Figure 4:**
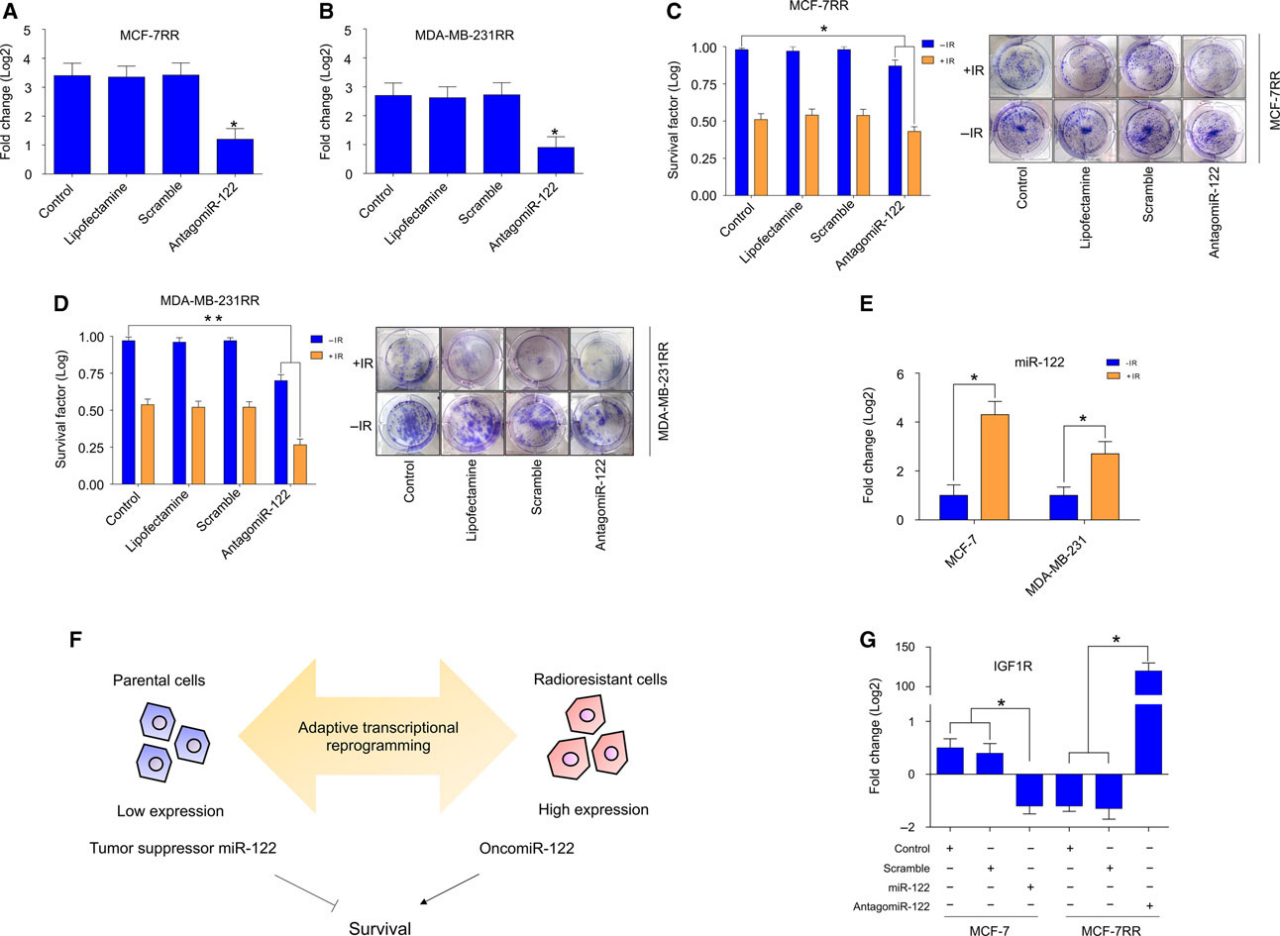
miR‐122 is overexpressed in radioresistant breast cancer cells and its inhibition reverts the radioresistant phenotype. Knockdown of miR‐122 in radioresistant (A) MCF‐7RR and (B) MDA‐MB‐231RR cells transfected with antagomiR‐122 was verified by qRT‐PCR assays. All values were normalized using RNU44 as an internal control. AntagomiR‐122‐transfected cells were evaluated for a radioresponse by clonogenic survival. Data for SF of transfected (C) MCF‐7RR and (D) MDA‐MB‐231RR cells irradiated (+IR) with 4 Gy of X‐ray are shown. Data were normalized using non‐irradiated cells (−IR). Representative images of the results of the clonogenic assays for MCF‐7RR and MDA‐MB‐231RR cells are shown in (C) and (D). (E) Overexpression of miR‐122 in parental MCF‐7 and MDA‐MB‐231 induced by treatment with 4 Gy of X‐ray was evaluated by qRT‐PCR assays. The expression data were normalized using parental MCF‐7 and MDA‐MB‐231 cells. All values were normalized using RNU44 as an internal control. (F) Schematic representation of the role of miR‐122 as a tumor suppressor miRNA in parental breast cancer cells and its oncogenic role during the transition from a cancer cell to a radioresistant cancer cell. (G) Expression of *IGF1R* in MCF‐7 and MCF‐7RR cells transfected with mimic‐miR122 and antagomiR‐122, respectively, was evaluated by qRT‐PCR. All values were normalized using GAPDH as an internal control. Data are presented graphically as the mean ± SD of three independent experiments. *P ≤ 0.05; **P < 0.01 by ANOVA.

We have demonstrated that miR‐122 is up‐regulated in both radioresistant breast tumor cells. To obtain additional insight into the biological function of miR‐122 in acquired radioresistance, we performed loss‐of‐function assays in MCF‐7RR and MDA‐MB‐231RR cells. We efficiently inhibited the expression of miR‐122 in MCF‐7RR (Fig. 4A) and MDA‐MB‐231RR (Fig. 4B) cells using antagomiR‐122. The transfected cells were evaluated for radioresistance using clonogenic assays. As expected, the radioresistant cell lines with a deficiency in miR‐122 had a significantly reduced survival efficiency. The results showed that knockdown of miR‐122 alone (non‐irradiated cells) but not in transfected cells with scrambled control had a significantly negative effect on survival rates in both MCF‐7RR (SF = 0.87) (Fig. 4C) and MDA‐MB‐231RR (SF = 0.70) (Fig. 4D). This negative effect on survival was higher when cells were irradiated (MCF‐7RR: SF = 0.43) (Fig. 4C) (MDA‐MB‐231RR: SF = 0.26) (Fig. 4D). Hence, the knockdown of miR‐122 in radiotherapy‐induced resistant cells is able to revert the radioresistance of the cells via delayed cell survival. Furthermore, these results suggest that miR‐122 promotes survival pathways with respect to maintaining a radioresistant phenotype in breast cancer cells. Accordingly, we evaluated the expression of miR‐122 in parental cells treated with radiotherapy. The results showed that radiation promotes overexpression of miR‐122 in parental breast cancer cells (Fig. 4E). We hypothesized that overexpression of miR‐122 was maintained during the adaptive biological reprogramming in response to continuous application of radiation (i.e. during the transition from a cancer cell to a radioresistant cancer cell) (Fig. 4F). Moreover, it is likely that miR‐122 could gain an oncogenic role in radioresistant cells, therefore having a dual function in breast cancer cells, acting either as a tumor suppressor or an oncogene depending on the cellular context (Fig. 4F). The adaptive transcriptional reprogramming includes the possibility that target genes of the miRNAs could be also changed, producing oncogenic or tumor‐suppressive effect. In this way, miR‐122 should regulate different target genes to act as a positive regulator of the survival pathways, thus favoring an oncogenic function in radioresistant cells. To explore this hypothesis, we evaluated the expression of *IGF1R*, which has previously been reported as a target gene of miR‐122 in breast cancer (Wang *et al*., 2012). Remarkably, the results obtained via qRT‐PCR showed that *IGF1R* is down‐regulated in parental MCF‐7 cells with gain‐of‐function of miR‐122 and up‐regulated in MCF‐7RR cells with loss‐of‐function of miR‐122 (Fig. 4G), which is consistent with targeted activity of miR‐122. These results suggest that radiosensitivity observed in MCF‐7RR cells with loss‐of‐function of miR‐122 could be independent of the *IGF1R* function. Taken together, these results indicate that miR‐122 has an oncogenic role in the acquired radioresistance of breast cancer cells.

### 3.5 Transcriptomic landscape of the radioresistant breast cancer cells with loss‐of‐function of miR‐122

**Figure 5:**
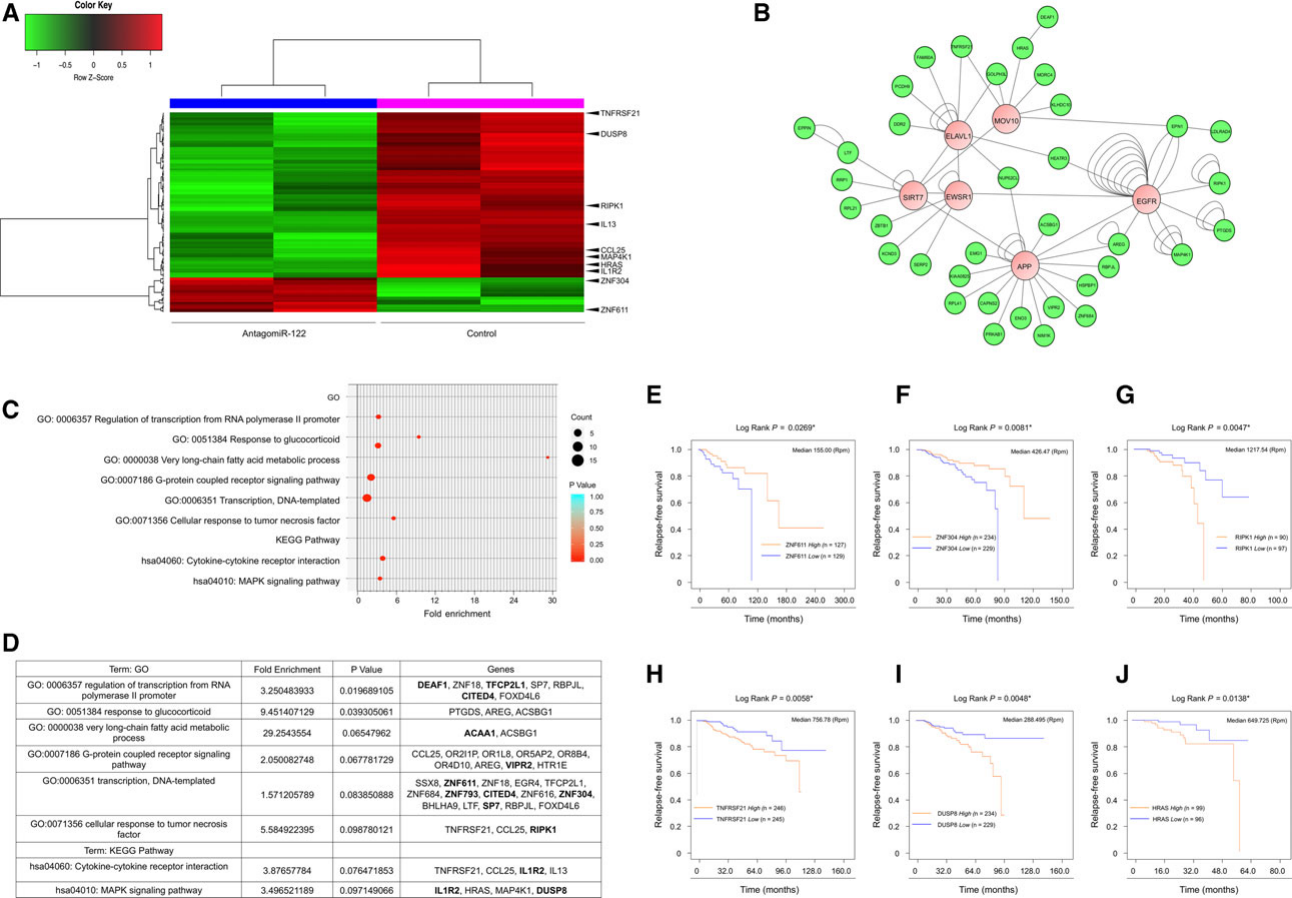
Transcriptome analysis of MCF‐7RR cells with knockdown of miR‐122 and analysis of prognostic factor genes in breast cancer patients. (A) Heat map showing the differential expression pattern of 158 genes in MCF‐7RR cells transfected with antangomiR‐122. The heat map indicates 131 up‐regulated (red) and 27 down‐regulated (green) genes. The columns represent a duplicate of individual samples of MCF‐7RR cells with knockdown of miR‐122, as well as MCF‐7RR untransfected cells. The rows represent individual genes. Arrows indicate genes with prognostic value in breast cancer patients treated with radiotherapy. (B) Interactome generated from protein–protein interaction data by Key Pathway cytoscape (Alcaraz *et al*., 2014). The 36 genes shown in green are the modulated genes by knockdown of miR‐122. The six genes shown in pink are linker genes that connect the 36 genes from transcriptome. The numbers of connections in the network are shown as nodes. (C) Bubble chart shows the enriched GO terms of the genes modulated by the knockdown of miR‐122. Biological processes are shown on the *y*‐axis. The color and size of the bubble represent the number of genes involved in each GO/network process and significance, respectively. (D) Chart of GO and biological processes of genes modulated by the knockdown of miR‐122. Genes symbol are shown. Bold‐labeling indicates genes containing miR‐122 binding sites in their 3′‐UTR. Kaplan–Meier curves of RFS. Survival curves of high vs low expression of (E) *ZNF611*, (F) *ZNF304*, (G) *RIPK1*, (H) *TNFRSF21*, (I) *DUSP8* and (J) *HRAS* of patients with breast cancer treated with radiotherapy. High or low gene expression levels according to > median or ≤ median expression levels each gene. Curves were compared using a log‐rank test *P ≤ 0.05; **P ≤ 0.01. Rpm, reads per million.

To obtain a comprehensive molecular understanding of the oncogenic role of miR‐122 in acquired radioresistance, we utilized a gene expression profiling approach with microarrays aiming to systematically identify genes associated with loss‐of‐function of miR‐122 in radioresistant cells. In total, 158 differentially expressed genes were identified in hierarchical clustering analysis (Fig. 5A). Twenty‐seven genes were up‐regulated (fold change ≥ 1.3) and 131 genes were down‐regulated (fold change ≤ −1.3); (Table S1). Using the Key Pathway Miner App of cytoscape to determine interconnected pathways by protein–protein interaction *in silico*, we revealed products of genes that were not observed in the microarrays assays (outliers), such as *EGFR*,* APP*,* MOV10*,* EWSR1*,* SIRT7* and *ELAVL1* (Fig. 5B). Our analysis by functional protein association networks, GO and biological pathways (Fig. 5C) showed that the gene signature in knockdown miR‐122‐MCF‐7RR could be associated with regulation of transcription (*SSX8*,* ZNF611*,* ZNF18*,* EGR4*,* TFCP2L1*,* ZNF684*,* ZNF793*,* CITED4*,* ZNF616*,* ZNF304*,* BHLHA9*,* LTF*,* SP7*,* RBPJL*,* FOXD4L6*), the G‐protein coupled receptor signaling pathway (*CCL25*,* OR2I1P*,* OR1L8*,* OR5AP2*,* OR8B4*,* OR4D10*,* AREG*,* VIPR2*,* HTR1E*), the TNF pathway (*TNFRSF21*,* CCL25*,* RIPK1*), the Ras‐MAPK pathway (*IL1R2*,* HRAS*,* MAP4K1*,* DUSP8*) and the inflammatory response (*TNFRSF21*,* CCL25*,* IL1R2*,* IL13*) (Fig. 5D). To identify potential genes that could be directly regulated by miR‐122, we performed *in silico* analysis of 3'‐UTR binding sites for miR‐122 in modulated genes. We found nine up‐regulated and 29 down‐regulated genes containing canonical 3′‐UTR binding sites for miR‐122 (Table 3), highlighting the up‐regulation of *IGLON5*,* NUP62CL*,* ACAA1*,* KLHL5*,* FBXO48*,* ZNF304*,* VIPR2*,* CCDC127* and *ZNF611*, as well as the down‐regulation of *DUSP8*,* DDR2*,* IL1R2*,* DEAF1* and *RIPK1*. Therefore, microarrays analysis revealed that radioresistant cells with loss‐of‐function of miR‐122 are highly enriched for genes encoding signaling pathways and transcriptional processes, suggesting that a major influence of miR‐122 function on acquired radioresistance is related to maintaining survival networks.

**Table 3:** Genes differentially regulated in knockdown miR‐122‐MCF‐7RR cells with a 3′‐UTR‐canonical binding site to miR‐122

Gene	Function
Up‐regulated	
*IGLON5*	Neuronal cell‐adhesion protein
*NUP62CL*	Nucleocytoplasmic transporter activity
*ACAA1*	Acetyl‐CoA C‐acyltransferase activity
*KLHL5*	Ubiquitin‐protein transferase activity
*FBXO48*	Decreased interleukin‐8 secretion
*ZNF304*	Activation of KRAS and silencing of several tumor suppressor genes
*VIPR2*	Pituitary adenylate cyclase activation
*CCDC127*	Cell surface transport
*ZNF611*	Transcriptional regulation
Down‐regulated	
*LCE2C*	Precursors of the cornified envelope of the stratum
*PRH2*	Protective and reparative environment for dental enamel
*RAB40AL*	Mediates the ubiquitination and subsequent proteasomal degradation of target proteins
*MICAL2*	Actin binding and flavin adenine dinucleotide binding
*SEMA5A*	Semaphorin gene family that encodes membrane proteins containing a semaphorin domain and several thrombospondin type‐1 repeats. May promote angiogenesis by increasing endothelial cell proliferation and migration and inhibiting apoptosis
*SLC25A18*	Involved in the transport of glutamate across the inner mitochondrial membrane
*PODNL1*	Small leucine‐rich repeat protein family
*DUSP8*	Phosphatase activity with synthetic phosphatase substrates and negatively regulates mitogen‐activated protein kinase activity
*DDR2*	Tyrosine kinase that functions as cell surface receptor for fibrillar collagen and regulates cell differentiation, remodeling of the extracellular matrix, cell migration and cell proliferation
*C2CD4A*	Involved in inflammatory process. May regulate cell architecture and adhesion
*KIAA0825*	Cell surface transport
*ZNF793*	Transcriptional regulation
*DLX4*	Play a role in determining the production of hemoglobin S. May act as a repressor
*LIN28A*	Protein that acts as a posttranscriptional regulator of genes involved in developmental timing and self‐renewal in embryonic stem cells. Disrupting the maturation of certain miRNAs
*SP7*	Transcriptional activator essential for osteoblast differentiation
*TFCP2L1*	Transcriptional suppressor, cellular self‐renewal
*CITED4*	Acts as transcriptional coactivator for TFAP2/AP‐2. Enhances estrogen‐dependent transactivation mediated by estrogen receptors
*TRPM1*	Cation channel essential for the depolarizing photoresponse of retinal ON bipolar cells. It is part of the GRM6 signaling cascade. Metastasis in melanoma
*IL1R2*	Cytokine receptor that belongs to the interleukin‐1 receptor family
*VEPH1*	Downregulation of Wnt pathway after Wnt3A stimulation
*ARHGAP23*	GTPase activator for the Rho‐type GTPases by converting them to an inactive GDP‐bound state
*C4B*	Complement component 4B, including the ZB transcript in the same orientation, complexing with C2 to form the C3/C5 convertase, classical pathway
*CNIH3*	Regulates the trafficking and gating properties of AMPA‐selective glutamate receptors
*DEAF1*	Cell proliferation, arresting cells in the G0 or G1 phase. Required for neural tube closure and skeletal patterning. Regulates epithelial cell proliferation and side‐branching in the mammary gland
*EPPIN*	Serine protease inhibitor that plays an essential role in male reproduction and fertility
*HEATR3*	Role in ribosomal protein transport and in the assembly of the 5S ribonucleoprotein particle (5S RNP). The encoded protein also may be involved in NOD2‐mediated NF‐kappa B signaling
*NUB1*	Protein that functions as a negative regulator of NEDD8, a ubiquitin‐like protein that conjugates with cullin family members in order to regulate vital biological events
*RIPK1*	Serine‐threonine kinase, which transduces inflammatory and cell‐death signals (programmed necrosis) following death receptors ligation, activation of pathogen recognition receptors and DNA damage. Activates the MAP3K5‐JNK apoptotic cascade
*KRT77*	Responsible for the structural integrity of epithelial cells

### 3.6 Genes associated with loss‐of‐function of miR‐122 are involved in the outcome of breast cancer patients treated with radiotherapy

To determine the prognostic value of genes associated with loss‐of‐function of miR‐122, we evaluated RFS in 491 breast cancer patients treated with radiotherapy, as obtained from the TCGA database. Patients were categorized according to the median expression of each gene; therefore, we obtained different groups of patients according to the low and high expression of an individual gene. Characteristic populations are shown in Tables S2–S7. In the analysis for RFS, differential expression of *ZNF611*,* ZNF304*,* RIPK1*,* DUSP8*,* TNFRSF21* and *HRAS* genes was associated with outcome for breast cancer patients who received radiotherapy. Kaplan–Meier curves showed that increased expression of *ZNF611* (*P*  = 0.0269; Fig. 5E) and *ZNF304* (*P* = 0.0081; Fig. 5F), as well as lower expression of *RIPK1* (*P* = 0.0047; Fig. 5G), *TNFRSF21* (*P* = 0.0058; Fig. 5H) and *DUSP8* (*P* = 0.048; Fig. 5I) and *HRAS* (*P* = 0.0138; Fig. 5J), was associated with longer RFS. These results were in accordance with the experimental evidence obtained in MCF‐7RR cells in which an increase of *ZNF611* and *ZNF304* expression, in addition to a decrease of *RIPK1*,* TNFRSF21*,* DUSP8* and *HRAS* expression, induced by knockdown of miR‐122 was correlated with radiosensitivity *in vitro*.

### 3.7 miR‐122 differentially controls levels of *ZNF611*,* ZNF304*,* RIPK1*,* DUSP8*,* HRAS* and *TNFRS21* protein in radioresistant breast cancer cells

**Figure 6:**
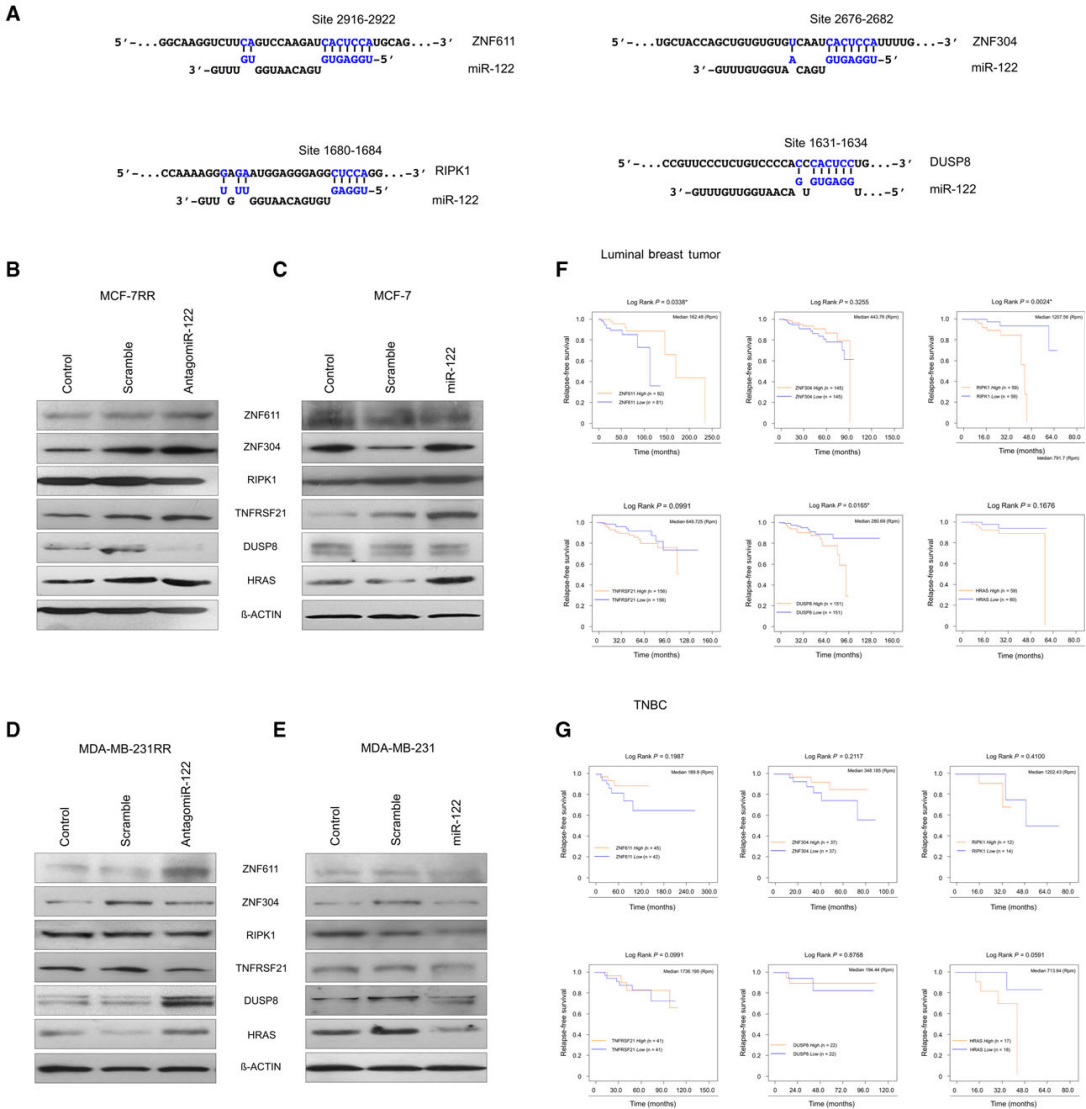
Levels of *ZNF611*,* ZNF304*,* RIPK1*,* TNFRSF21*,* DUSP8* and *HRAS* are differentially regulated by miR‐122 in parental and radioresistant breast cancer cells. (A) Schematic illustration of the potential miR‐122 binding site in the 3′‐UTR of *ZNF611*,* ZNF304*,* RIPK1* and *DUSP8* genes. Letters labeled in blue indicate the seed region. Levels of *ZNF611*,* ZNF304*,* RIPK1*,* TNFRSF21*,* DUSP8* and *HRAS* proteins were evaluated by western blot assays in (B) MCF‐7RR transfected with antagomiR‐122. (C) Parental MCF‐7 transfected with mimic‐miR122. (D) MDA‐MB‐231RR transfected with antagomiR‐122. (E) Parental MDA‐MB‐231 transfected with mimic‐miR122. β‐actin was used as an internal control. Images are representative of three independent experiments. Kaplan–Meier curves of RFS. Survival curves of high vs low expression of *ZNF611*,* ZNF304*,* RIPK1*,* TNFRSF21*,* DUSP8* and *HRAS* of patients with (F) luminal breast cancer and (G) TNBC treated with radiotherapy. High or low gene expression levels according to > median or ≤ median expression levels each gene. Curves were compared using a log‐rank test *P ≤ 0.05.

Our findings obtained by Kaplan–Meier analysis revealed that *ZNF611*,* ZNF304*,* RIPK1*,* DUSP8*,* TNFRSF21* and *HRAS* genes have a prognostic value in patients treated with radiotherapy. Among these genes, *ZNF611* (positions 2916–2922), *ZNF304* (positions 2676–2682), *RIPK1* (positions 1680–1684) and *DUSP8* (positions 1631–1634) contain canonical miR‐122 3′ UTR‐binding sites (Fig. 6A). For experimental validation of the transcriptome results, and also to test whether modulation of *ZNF611*,* ZNF304*,* RIPK1*,* DUSP8*,* HRAS* and *TNFRS21* following knockdown of miR‐122 might be a common event in radioresistant breast cancer cells, we performed western blot assays in transfected MCF‐7RR and MDA‐MB‐231RR cells with antagomiR‐122. As expected, we verified the results obtained by microarrays assays in MCF‐7RR cells. Levels of proteins *ZNF304* and *ZNF611* were up‐regulated, whereas *RIPK1* and *DUSP8* were repressed, when we inhibited miR‐122 (Fig. 6B). Levels of *HRAS* and *TNFRS21* levels were observed with non‐significant changes. The results showed a direct correlation among the expression of miR‐122 and *ZNF304*,* ZNF611*,* RIPK1* and *DUSP8* levels. As a control, we compared the abundance of these proteins in parental MCF‐7 cells transfected with mimic‐miR122 (Fig. 6C). The results showed that the expression of *ZNF304*,* RIPK1*,* DUSP8* and *TNFRS21* was not changed when we forced overexpression of miR‐122, whereas the expression of *HRAS* was higher compared to non‐transfected and scrambled transfected cells (Fig. 6C). Notably, *ZNF611* was down‐regulated when miR‐122 was overexpressed (Fig. 6C). These findings suggested that miR‐122 could target *ZNF611* but was unable to modulate *ZNF304*,* RIPK1*,* DUSP8* and *TNFRS21* protein levels in parental MCF‐7 cells. Although not all of the western blot results were similar in MDA‐MB‐231RR compared to MCF‐7RR, we observed that, in MDA‐MB‐231RR cells, knockdown of miR‐122 was correlated with up‐regulation of *ZNF611*,* DUSP8* and *HRAS* (Fig. 6D). Moreover, in parental MDA‐MB‐231 cells, forced overexpression of miR‐122 was also correlated with inhibition of *ZNF611*,* DUSP8* and *HRAS* levels (Fig. 6E). These results suggested that *ZNF611* and *DUSP8* might be targeted by miR‐122 in the MDA‐MB‐231RR model. It should be noted that miR‐122 could modulate dissimilar pathways in radioresistant breast cancer cells compared to parental breast cancer cells, which might partially explain the dual function as a tumor suppressor or oncomiR depending on the cellular context. On the other hand, the variability of the results among the radioresistant cells could be a result of a difference in cellular context with respect to the origin of the cell lines because MDA‐MB‐231 is a model of the triple‐negative breast cancer (TNBC) subtype, whereas MCF‐7 is a model of a luminal tumor. In this sense, we evaluated the prognostic value of the *ZNF611*,* ZNF304*,* RIPK1*,* TNFRSF21*,* DUSP8* and *HRAS* genes according to tumor subtypes. The RFS of patients with luminal breast cancer and TNBC treated with radiotherapy was evaluated by Kaplan–Meier analysis. The results showed that higher levels of *ZNF611* (*P* = 0.0338), as well as lower levels of *RIPK1* (*P* = 0.0024) and *DUSP8* (*P* = 0.0165), were associated with longer RFS in luminal subtypes (Fig. 6F). However, although these genes were not significantly associated with RFS in TNBC subtypes, higher levels of ZNF611 and ZNF304 and lower levels of HRAS showed a trend for an association with a longer RFS (Fig. 6G).

## 4 Discussion

To gain insight into the molecular adaptations underpinning the radioresistance of breast cancer cells, we report the development of isogenic radioresistant breast cancer cell lines, miRNome landscape analysis and functional analysis of miR‐122. An isogenic model of radioresistance was developed by cumulative exposure of MCF‐7 and MDA‐MB‐231 cells to 30 Gy‐fractionated radiation, resulting in the generation of a subline with significantly increased survival potential compared to sham control cells, as reported previously in a model of radioresistant lung cancer cells (Arechaga‐Ocampo *et al*., 2017). The development of isogenic chemo‐ and radioresistant cell lines has been used successfully to investigate the molecular changes associated with acquired resistance to therapy and tumor aggressiveness in cancer (McDermott *et al*., 2014). Exposure of tumors to fractionated radiation schedules can select a cancer cell subpopulation with an increased capacity to overcome the anti‐proliferative effects of radiotherapy (Zaider and Hanin, 2011) by modulating the abundance and functions of molecules, including miRNAs (Arechaga‐Ocampo *et al*., 2017; Metheetrairut and Slack, 2013; Zhang *et al*., 2014). In the present study, we identified a set of miRNAs related to acquired radioresistance. Wang *et al*. (2014b) have shown that the expression of a group of miRNAs establishes a useful molecular signature with respect to differentiating radioresistant from non‐radioresistant tumors, even though they originate from the same histological type. In this sense, we identified the miRNome of the isogenic MCF‐7RR cell line compared to the parental MCF‐7 cell line and found 16 miRNAs to be differentially expressed (13 up‐ and three down‐regulated). Among them, miR‐184 (Fang *et al*., 2017), miR‐424* (Zhang *et al*., 2017b), miR‐218 (Wang *et al*., 2017), miR‐222 (Wei *et al*., 2017) and miR‐10a (Rong *et al*., 2016) have been reported in relation to chemotherapy resistance in several types of tumors. Others, such as as miR‐135b* (Wang *et al*., 2014a,2014b), miR‐223* (de Melo Maia *et al*., 2016), miR‐135b (Han *et al*., 2017) and miR‐196b (Ren *et al*., 2017), have been reported as oncomiRs. The aberrant expression of miRNAs in the radioresistance of human tumors is a result of their function as negative regulators of gene expression. miRNAs can control the expression of genes that are components of cell survival pathways, apoptosis, the immune response and cell differentiation, amongst others (Bartel, 2004). The results of our bioinformatic analysis of the GO and biological pathways revealed that the set of miRNAs is implicated in proliferation and survival pathways, the immune response and transcriptional control, suggesting that signaling pathways involved in acquired radioresistance can be directed by the coordinated action of these molecules. It is remarkable that deregulation of the miRNAs identified in MCF‐7RR cells is conserved in MDA‐MB‐231RR cells. Specially, miR‐196b, miR‐222 and miR‐122 show similar expression in both radioresistant cell lines, which suggests that they might have a significant role in the phenotypic evolution of cancer cells to acquired radioresistance. Notably, miR‐122 is known to act as a tumor suppressor in breast cancer by targeting *IGF1R* (Wang *et al*., 2012) and *ADAM10* genes (Ergün *et al*., 2015). In addition, miR‐122 has the same role in liver and glioma tumors, in which it suppresses mechanisms to promote tumor progression and survival (Wang *et al*., 2014a,2014b). In the present study, we demonstrate that miR‐122 significantly reduced the survival of the parental cells, although this effect was enhanced when the cells were irradiated. This result is consistent with previous reports of the tumor suppressor function of miR‐122 in breast cancer (Ergün *et al*., 2015; Wang *et al*., 2012) and it also highlights its potential as a radiosensitizer. Similarly, a previous study showed that miR‐122 induces radiosensitivity in lung cancer cells exposed to different doses of radiation. The ectopic overexpression of miR‐122 in lung cancer cells decreased anchorage‐dependent invasion and inhibited cell growth by knockdown of target genes related to tumor survival and the cellular stress response (Ma *et al*., 2015). The role of miR‐122 as a radiosensitizer in lung cancer is consistent with our findings in breast cancer. Recently Zhang *et al*. (2017a,2017b) reported the function of miR‐122‐3p in response to radiation in TNBC cells. It was demonstrated that miR‐122‐3p promotes sensitivity to radiation by modifying cellular apoptosis, migration and invasion as a result of modulation of the PTEN/PI3K/AKT pathway. It should be noted that miR‐122‐3p (miR‐122*) is the passenger strand of leading strand miR‐122‐5p (miR‐122); therefore, miR‐122‐3p could target different genes, giving it an independent function compared to miR‐122‐5p. The results of the studies by Zhang *et al*. (2017a,2017b), together with our results, suggest that miR‐122* and miR‐122 might function cooperatively to promote the sensitivity of breast cancer cells exposed to radiation. However, in our isogenic model of acquired radioresistant breast cancer cells, we did not observed the aberrant expression of miR‐122‐3p and so the role of miR‐122‐3p in acquired radioresistance in breast cancer still remains unknown.

Besides its function *in vitro,* we show that the expression of miR‐122 in breast tumors was significantly associated with a favorable response to radiotherapy compared to those patients who did not express miR‐122. In such patients, miR‐122 has a tumor‐suppressive role for treatment‐naïve tumors (i.e. in patients who have not yet received radiation therapy and therefore radioresistance has not yet developed). With these results, we demonstrate that miR‐122 is related to the sensitivity to radiotherapy in parental breast cancer cells both *in vitro* and *in vivo* in a set of breast tumors, which, to our knowledge, has not been reported previously. Unexpectedly, in MCF‐7RR and MDA‐MB‐231RR cells, we observed overexpression of miR‐122. Its downregulation by antagomiR‐122 was able to revert the resistance to radiotherapy by counteracting cell survival. By contrast to functioning as a tumor suppressor, miR‐122 appears to have an oncogenic role in breast cancer cells that have acquired radioresistance. It is known that miRNAs can act as a tumor suppressor or as an oncogene depending on the scenario. Svoronos *et al*. (2016) provide an excellent review in which they discuss the dual role of miRNAs in cancer cells themselves and as extrinsic factors. They also report that dual function could be dependent on different cell phenotypes, tumor microenvironment, immune evasion and by selective pressure induced by therapy treatments, including radiotherapy. We showed that miR‐122 is up‐regulated in response to radiation treatment, suggesting that continuous fractionated irradiation maintains the overexpression of miR‐122 until a radioresistant phenotype is attained. Based on our results, we propose that miR‐122 could acquire an oncogenic function under the pressure exerted by radiation in breast cancer, which allows evolution of the cell and adaption to radiotherapy. To explore the genes and biological pathways modulated by miR‐122 in radioresistant cells, we analyzed the transcriptome of MCF‐7RR cells with loss‐of‐function of miR‐122. The results showed 158 genes to be differentially modulated, of which 27 were increased and 131 were decreased. Analyses of the biological network by cytoscape revealed protein outliers that have been reported in radiotherapy resistance in cancer and survival, as well as proliferation and epigenetic regulation, such as *EGFR* (Lee *et al*., 2011), *MOV10* (El Messaoudi‐Aubert *et al*., 2010), *ELAVL1* (Mehta *et al*., 2016), *SIRT7* (Chen *et al*., 2017; Tang *et al*., 2017), *APP* (Lim *et al*., 2014) and *EWSR1* (Suzuki *et al*., 2012). Many studies have reported that radiotherapy can induce transcriptional reprogramming to enable acquired resistance and to avoid the toxicity triggered by radiotherapy (Doan *et al*., 2018; Ma *et al*., 2013). We determined that miR‐122 modulates genes related to molecular processes such as transcriptional regulation and the signaling of receptors coupled to G proteins, as well as the MAPK and TNF pathways. This diversity of cellular processes is consistent with numerous regulatory mechanisms associated with the response to radiation and radioresistance in breast cancer (Kaidar‐Person *et al*., 2013). In the transcriptomic landscape induced by knockdown miR‐122, we identified genes involved in these cellular processes such as *ZNF611*,* ZNF304*,* RIPK1*,* DUSP8*,* HRAS* and *TNFRSF21*. Moreover, these genes were prognostic factors in breast cancer patients treated with radiotherapy. The clinical findings were in accordance with the results of survival assays *in vitro*. Among the set of up‐regulated genes, we found that *ZNF611* and *ZNF304* contain canonical miR‐122‐binding sites in their 3′‐UTR region; in addition, we verified that the protein levels of these transcription factors also increase when miR‐122 is inhibited in MCF‐7RR cells. *ZNF304* and *ZNF611* are transcription factors that belong to the C2H2 zinc finger family possessing Krüppel associated box and are related to transcriptional silencing by the recruitment of epigenetic complexes (Aslan *et al*., 2015; Pengue and Lania, 1996). ZNF304 has also been reported as a regulator of the RAS pathway by recruitment of an epigenetic silencing complex in tumor suppressor genes in colorectal cancer (Serra *et al*., 2014) and as a promoter of cell survival in ovarian cancer (Aslan *et al*., 2015). ZNF611 has not yet been reported in cancer. On the other hand, *RIPK1*,* DUSP8*,* HRAS* and *TNFRS21* were down‐regulated when we knocked‐down miR‐122. *RIPK1* (Miyamoto, 2011) and *TNFRS21* (Benschop *et al*., 2009) are components of the TNF pathway, whereas *DUSP8* (Keyse, 2008) and *HRAS* (Knobbe *et al*., 2004) act on the RAS‐MAPK pathway. Alteration in the activity of transcription factors could be critical for the development and maintenance of radioresistance in breast cancer by control genes of the survival pathways, such as the RAS‐MAPK and TNF pathways (Ishihara *et al*., 2015; Zhao *et al*., 2018). Many of the genes that we observed to be down‐regulated in the transcriptome analysis might be part of an epigenetic network of transcriptional silencing driven by axis miR‐122‐*ZNF611* or miR‐122‐*ZNF304*. This is a research topic that is currently under investigation; however, we have obtained preliminary data strongly suggesting that *RIPK1* and *DUSP8* possess elements of response to ZNF611 and ZNF304 in their promoter regions (data not shown). Therefore, we propose that the dual function of miR‐122 in the isogenic model of radioresistant breast cancer cells could be result of transcriptional reprogramming controlled by the modulation of *ZNF611* and *ZNF304* by miR‐122.

## 5 Conclusions

**Figure 7:**
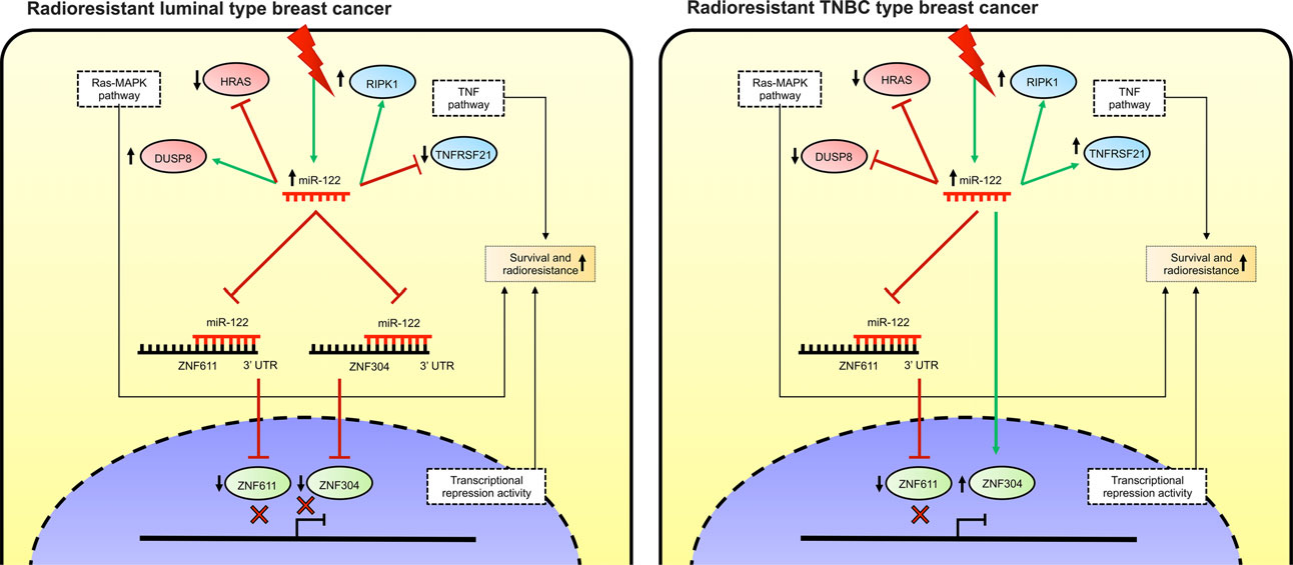
Schematic representation of miR‐122‐mediated radioresistance in breast cancer cells. miR‐122 has an oncogenic role in the acquired radioresistance of luminal and TNBC cells by differentially controlling the expression of ZNF611 and ZNF304 transcription factors, as well as modulating the expression of genes involved in RAS‐MAPK and TNF pathways to promote survival.

In conclusion, the data obtained in the present study contribute to our understanding of the mechanisms of molecular adaptation to radiotherapy in breast cancer cells. The evidence indicates the aberrant expression of a set of miRNAs linked to carcinogenesis and the molecular control of pathways related to the response to therapy in cancer. Particularly, the overexpression of miR‐122 maintains the radioresistant phenotype in breast cancer cells by promoting cell survival, from the regulation of several genes to the downstream effects of these genes, which confers it with an oncogenic function (Fig. 7).

## Conflict of interests

The authors declare no conflict of interest.

## Author contributions

IXP‐A, EA‐O and CHG‐DR were responsible for study conception and design. IXP‐A, EA‐O, CHGD‐R and FOB‐A were responsible for development of the methodology. IXP‐A, EA‐O, FOB‐A, MS‐A and OA‐Z were responsible for the acquisition of data. IXP‐A, EA‐O, CHGD‐R, ES‐R and FOB‐A were responsible for the analysis and interpretation of data. IXP‐A, EA‐O, CHGD‐R, ES‐R, FOB‐A, DAL‐H and MC‐S were responsible for writing, reviewing and/or revising the manuscript. EA‐O, NV‐S, JAG‐B, CHGD‐R, ODM‐H, ES‐R and AG‐C were responsible for administrative, technical or material support. IXP‐A, EA‐O, CHGD‐R and ES‐R were responsible for study supervision.

## Supporting information


**Fig. S1.** Validation of the expression of a set of miRNAs deregulated in MCF‐7RR cells. Expression of a set of miRNAs was evaluated by qRT‐PCR in MCF‐7RR cells. The expression data were normalized using the parental MCF‐7 cells. All values were normalized using RNU44 as an internal control. Data for the qRT‐PCR assays were compared with the results from global expression profiles by the TLDAs system. Data are presented as the mean ± SD of three independent experiments. *P < 0.01 by Student's *t*‐test.

**Table S1.** Differentially expressed genes in the MCF‐7RR cells transfected with antagomiR‐122.

**Table S2.** Characteristics of the population of breast cancer patients based on expression of the *ZNF611* gene.
**Table S3.** Characteristics of the population of breast cancer patients based on expression of the *ZNF304* gene.
**Table S4.** Characteristics of the population of breast cancer patients based on expression of the *RIPK1* gene.
**Table S5.** Characteristics of the population of breast cancer patients based on expression of the *TNFRSF21* gene.
**Table S6.** Characteristics of the population of breast cancer patients based on expression of the *DUSP8* gene.
**Table S7.** Characteristics of the population of breast cancer patients based on expression of the *HRAS* gene.

## Data Availability

Microarray raw data tables have been deposited at the National Center for Biotechnology Information Gene Expression Omnibus (https://www.ncbi.nlm.nih.gov/geo/query/acc.cgi?acc=GSE120171).

## References

[mol212483-bib-0001] Alcaraz N , Pauling J , Batra R , Barbosa E , Junge A , Christensen AG , Azevedo V , Ditzel HJ and Baumbach J (2014) Key Pathway Miner 4.0: condition‐specific pathway analysis by combining multiple omics studies and networks with Cytoscape. BMC Syst Biol 8, 99. 25134827 10.1186/s12918-014-0099-x PMC4236746

[mol212483-bib-0002] Arechaga‐Ocampo E , Lopez‐Camarillo C , Villegas‐Sepulveda N , Gonzalez‐De la Rosa CH , Perez‐Añorve IX , Roldan‐Perez R , Flores‐Perez A , Peña‐Curiel O , Angeles‐Zaragoza O , Rangel Corona R *et al* (2017) Tumor suppressor miR‐29c regulates radioresistance in lung cancer cells. Tumour Biol 39, 3. 10.1177/1010428317695010 28345453

[mol212483-bib-0003] Aslan B , Monroig P , Hsu MC , Pena GA , Rodriguez‐Aguayo C , Gonzalez‐Villasana V , Rupaimoole R , Nagaraja AS , Mangala S , Han HD *et al* (2015) The ZNF304‐integrin axis protects against anoikis in cancer. Nat Commun 6, 7351. 26081979 10.1038/ncomms8351 PMC4830335

[mol212483-bib-0004] Bartel DP (2004) MicroRNAs: genomics, biogenesis, mechanism, and function. Cell 116, 281–297. 14744438 10.1016/s0092-8674(04)00045-5

[mol212483-bib-0005] Benschop R , Wei T and Na S (2009) Tumor necrosis factor receptor superfamily member 21: TNFR‐related death receptor‐6, DR6. Adv Exp Med Biol 647, 186–194. 19760075 10.1007/978-0-387-89520-8_13

[mol212483-bib-0006] Chen KL , Li L , Yang FX , Li CM , Wang YR and Wang GL (2017) SIRT7 depletion inhibits cell proliferation, migration and increases drug sensitivity by activating p38MAPK in breast cancer cells. J Cell Physiol 233, 6767–6778. 10.1002/jcp.26398 PMC13482412 29231244

[mol212483-bib-0007] Doan NB , Nguyen HS , Alhajala HS , Jaber B , Al‐Gizawiy MM , Ahn EE , Mueller WM , Chitambar CR , Mirza SP and Schmainda KM (2018) Identification of radiation responsive genes and transcriptome profiling via complete RNA sequencing in a stable radioresistant U87 glioblastoma model. Oncotarget 9, 23532–23542. 29805753 10.18632/oncotarget.25247 PMC5955095

[mol212483-bib-0008] El Messaoudi‐Aubert S , Nicholls J , Maertens GN , Brookes S , Bernstein E and Peters G (2010) Role for the MOV10 RNA helicase in polycomb‐mediated repression of the INK4a tumor suppressor. Nat Struct Mol Biol 17, 862–868. 20543829 10.1038/nsmb.1824 PMC2929459

[mol212483-bib-0009] Ergün S , Ulasli M , Igci YZ , Igci M , Kırkbes S , Borazan E , Balik A , Yumrutaş Ö , Camci C , Cakmak EA *et al* (2015) The association of the expression of miR‐122‐5p and its target ADAM10 with human breast cancer. Mol Biol Rep 42, 497–505. 25318895 10.1007/s11033-014-3793-2

[mol212483-bib-0010] Fang Z , Zhao J , Xie W , Sun Q , Wang H and Qiao B (2017) LncRNA UCA1 promotes proliferation and cisplatin resistance of oral squamous cell carcinoma by suppressing miR‐184 expression. Cancer Med 6, 2897–2908. 29125238 10.1002/cam4.1253 PMC5727307

[mol212483-bib-0011] Franken NA , Rodermond HM , Stap J , Haveman J and van Bree C (2006) Clonogenic assay of cells in vitro. Nat Protoc 1, 2315–2319. 17406473 10.1038/nprot.2006.339

[mol212483-bib-0012] Han X , Saiyin H , Zhao J , Fang Y , Rong Y , Shi C , Lou W and Kuang T (2017) Overexpression of miR‐135b‐5p promotes unfavorable clinical characteristics and poor prognosis via the repression of SFRP4 in pancreatic cancer. Oncotarget 8, 62195–62207. 28977937 10.18632/oncotarget.19150 PMC5617497

[mol212483-bib-0013] Ishihara S , Yasuda M , Ishizu A , Ishikawa M , Shirato H and Haga H (2015) Activating transcription factor 5 enhances radioresistance and malignancy in cancer cells. Oncotarget 6, 4602–4614. 25682872 10.18632/oncotarget.2912 PMC4467102

[mol212483-bib-0014] Jameel JK , Rao VS , Cawkwell L and Drew PJ (2004) Radioresistance in carcinoma of the breast. Breast 13, 452–460. 15563851 10.1016/j.breast.2004.08.004

[mol212483-bib-0015] Kaidar‐Person O , Lai C , Kuten A , Belkacemi Y and AROME (2013) “The Infinite Maze” of breast cancer, signaling pathways and radioresistance. Breast 22, 411–4118. 23642528 10.1016/j.breast.2013.04.003

[mol212483-bib-0016] Keyse SM (2008) Dual‐specificity MAP kinase phosphatases (MKPs) and cancer. Cancer Metastasis Rev 27, 253–261. 18330678 10.1007/s10555-008-9123-1

[mol212483-bib-0017] Knobbe CB , Reifenberger J and ReifenbeAslarger G (2004) Mutation analysis of the Ras pathway genes NRAS, HRAS, KRAS and BRAF in glioblastomas. Acta Neuropathol 108, 467–470. 15517309 10.1007/s00401-004-0929-9

[mol212483-bib-0018] Lee KM , Choi EJ and Kim IA (2011) microRNA‐7 increases radiosensitivity of human cancer cells with activated EGFR‐associated signaling. Radiother Oncol 101, 171–176. 21676478 10.1016/j.radonc.2011.05.050

[mol212483-bib-0019] Lim S , Yoo BK , Kim HS , Gilmore HL , Lee Y , Lee HP , Kim SJ , Letterio J and Lee HG (2014) Amyloid‐β precursor protein promotes cell proliferation and motility of advanced breast cancer. BMC Cancer 14, 928. 25491510 10.1186/1471-2407-14-928 PMC4295427

[mol212483-bib-0020] Ma D , Jia H , Qin M , Dai W , Wang T , Liang E , Dong G , Wang Z , Zhang Z and Feng F (2015) MiR‐122 induces radiosensitization in non‐small cell lung cancer cell line. Int J Mol Sci 9, 22137–22150. 10.3390/ijms160922137 PMC4613300 26389880

[mol212483-bib-0021] Ma H , Rao L , Wang HL , Mao ZW , Lei RH , Yang ZY , Qing H and Deng YL (2013) Transcriptome analysis of glioma cells for the dynamic response to γ‐irradiation and dual regulation of apoptosis genes: a new insight into radiotherapy for glioblastomas. Cell Death Dis 4, e895. 24176853 10.1038/cddis.2013.412 PMC3920930

[mol212483-bib-0022] McDermott N , Meunier A , Lynch TH , Hollywood D and Marignol L (2014) Isogenic radiation resistant cell lines: development and validation strategies. Int J Radiat Biol 90, 115–126. 24350914 10.3109/09553002.2014.873557

[mol212483-bib-0023] Mehta M , Basalingappa K , Griffith JN , Andrade D , Babu A , Amreddy N , Muralidharan R , Gorospe M , Herman T , Ding WQ *et al* (2016) HuR silencing elicits oxidative stress and DNA damage and sensitizes human triple‐negative breast cancer cells to radiotherapy. Oncotarget 40, 64820–64835. 10.18632/oncotarget.11706 PMC5323119 27588488

[mol212483-bib-0024] de Melo Maia B , Rodrigues IS , Akagi EM , Soares do Amaral N , Ling H , Monroig P , Soares FA , Calin GA and Rocha RM (2016) MiR‐223‐5p works as an oncomiR in vulvar carcinoma by TP63 suppression. Oncotarget 7, 49217–49231. 27359057 10.18632/oncotarget.10247 PMC5226502

[mol212483-bib-0025] Metheetrairut C and Slack FJ (2013) MicroRNAs in the ionizing radiation response and in radiotherapy. Curr Opin Genet Dev 1, 12–19. 10.1016/j.gde.2013.01.002 PMC3617065 23453900

[mol212483-bib-0026] Miyamoto S (2011) Nuclear initiated NF‐κB signaling: NEMO and ATM take center stage. Cell Res 21, 116–130. 21187855 10.1038/cr.2010.179 PMC3193401

[mol212483-bib-0027] Moran MS and Haffty BG (2002) Local‐regional breast cancer recurrence: prognostic groups based on patterns of failure. Breast J 8, 81–87. 11896752 10.1046/j.1524-4741.2002.08202.x

[mol212483-bib-0028] Pengue G and Lania L (1996) Krüppel‐associated box‐mediated repression of RNA polymerase II promoters is influenced by the arrangement of basal promoter elements. Proc Natl Acad Sci USA 93, 1015–1020. 8577706 10.1073/pnas.93.3.1015 PMC40022

[mol212483-bib-0029] Ren D , Lin B , Zhang X , Peng Y , Ye Z , Ma Y , Liang Y , Cao L , Li X , Li R *et al* (2017) Maintenance of cancer stemness by miR‐196b‐5p contributes to chemoresistance of colorectal cancer cells via activating STAT3 signaling pathway. Oncotarget 8, 49807–49823. 28591704 10.18632/oncotarget.17971 PMC5564809

[mol212483-bib-0030] Rong Y , Yuan CH , Qu Z , Zhou H , Guan Q , Yang N , Leng XH , Bu L , Wu K and Wang FB (2016) Doxorubicin resistant cancer cells activate myeloid‐derived suppressor cells by releasing PGE2. Sci Rep 6, 23824. 27032536 10.1038/srep23824 PMC4817121

[mol212483-bib-0031] Serra RW , Fang M , Park SM , Hutchinson L and Green MR (2014) A KRAS‐directed transcriptional silencing pathway that mediates the CpG island methylator phenotype. Elife 3, e02313. 24623306 10.7554/eLife.02313 PMC3949416

[mol212483-bib-0032] Suzuki K , Matsui Y , Higashimoto M , Kawaguchi Y , Seki S , Motomura H , Hori T , Yahara Y , Kanamori M and Kimura T (2012) Myxoid liposarcoma‐associated EWSR1‐DDIT3 selectively represses osteoblastic and chondrocytic transcription in multipotent mesenchymal cells. PLoS One 7, e36682. 22570737 10.1371/journal.pone.0036682 PMC3343026

[mol212483-bib-0033] Svoronos AA , Engelman DM and Slack FJ (2016) OncomiR or tumor suppressor? The duplicity of microRNAs in cancer. Cancer Res 1, 3666–3670. 10.1158/0008-5472.CAN-16-0359 PMC4930690 27325641

[mol212483-bib-0034] Tang M , Lu X , Zhang C , Du C , Cao L , Hou T , Li Z , Tu B , Cao Z , Li Y *et al* (2017) Downregulation of SIRT7 by 5‐fluorouracil induces radiosensitivity in human colorectal cancer. Theranostics 7, 1346–1359. 28435470 10.7150/thno.18804 PMC5399598

[mol212483-bib-0035] Torres‐Roca JF , Fulp WJ , Caudell JJ , Servant N , Bollet MA , van de Vijver M , Naghavi AO , Harris EE and Eschrich SA (2015) Integration of a radiosensitivity molecular signature into the assessment of local recurrence risk in breast cancer. Int J Radiat Oncol Biol Phys 93, 631–638. 26461005 10.1016/j.ijrobp.2015.06.021 PMC5811194

[mol212483-bib-0036] Wang B , Wang H and Yang Z (2012) MiR‐122 inhibits cell proliferation and tumorigenesis of breast cancer by targeting IGF1R. PLoS One 7, e47053. 23056576 10.1371/journal.pone.0047053 PMC3466252

[mol212483-bib-0037] Wang H , Zhan M , Xu SW , Chen W , Long MM , Shi YH , Liu Q , Mohan M and Wang J (2017) miR‐218‐5p restores sensitivity to gemcitabine through PRKCE/MDR1 axis in gallbladder cancer. Cell Death Dis 8, e2770. 28492560 10.1038/cddis.2017.178 PMC5520703

[mol212483-bib-0038] Wang G , Zhao Y and Zheng Y (2014a) MiR‐122/Wnt/β‐catenin regulatory circuitry sustains glioma progression. Tumour Biol 35, 8565–8572. 24863942 10.1007/s13277-014-2089-4

[mol212483-bib-0039] Wang L , Zhu MJ , Ren AM , Wu HF , Han WM , Tan RY and Tu RQ (2014b) A ten‐microRNA signature identified from a genome‐wide microRNA expression profiling in human epithelial ovarian cancer. PLoS One 9, e96472. 24816756 10.1371/journal.pone.0096472 PMC4015980

[mol212483-bib-0040] Wei F , Ma C , Zhou T , Dong X , Luo Q , Geng L , Ding L , Zhang Y , Zhang L , Li N *et al* (2017) Exosomes derived from gemcitabine‐resistant cells transfer malignant phenotypic traits via delivery of miRNA‐222‐3p. Mol Cancer 16, 132. 28743280 10.1186/s12943-017-0694-8 PMC5526308

[mol212483-bib-0041] Zaider M and Hanin L (2011) Tumor control probability in radiation treatment. Med Phys 38, 574–583. 21452694 10.1118/1.3521406

[mol212483-bib-0042] Zhang J , Cui Y , Lin X , Zhang Z and Li Z (2017a) MiR‐122‐3p sensitizes breast cancer cells to ionizing radiation via controlling of cell apoptosis, migration and invasion. Int J Clin Exp Pathol 10, 215–223.

[mol212483-bib-0043] Zhang P , Wang L , Rodriguez‐Aguayo C , Yuan Y , Debeb BG , Chen D , Sun Y , You MJ , Liu Y , Dean DC *et al* (2014) miR‐205 acts as a tumour radiosensitizer by targeting ZEB1 and Ubc13. Nat Commun 5, 5671. 25476932 10.1038/ncomms6671 PMC4377070

[mol212483-bib-0044] Zhang M , Zeng J , Zhao Z and Liu Z (2017b) Loss of MiR‐424‐3p, not miR‐424‐5p, confers chemoresistance through targeting YAP1 in non‐small cell lung cancer. Mol Carcinog 56, 821–832. 27500472 10.1002/mc.22536

[mol212483-bib-0045] Zhao W , Sun M , Li S , Chen Z and Geng D (2018) Transcription factor ATF3 mediates the radioresistance of breast cancer. J Cell Mol Med 22, 4664–4675. 30117642 10.1111/jcmm.13688 PMC6156394

