# Supplementary figures and images for: New insights into radioresistance in breast cancer identify a dual function of miR‐122 as a tumor suppressor and oncomiR
Source: Mol Oncol. 2019 Apr 18;13(5):1249–67. doi: 10.1002/1878-0261.12483 (PMC6487688; doi:10.1002/1878-0261.12483)

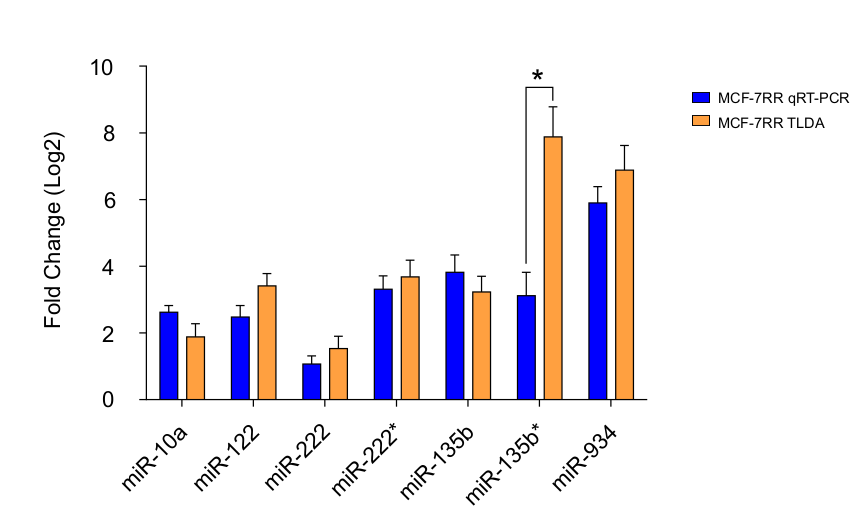

Supplement: Supplementary file 1 — Fig. S1. Validation of the expression of a set of miRNAs deregulated in MCF‐7RR cells. Expression of a set of miRNAs was evaluated by qRT‐PCR in MCF‐7RR cells. The expression data were normalized using the parental MCF‐7 cells. All values were normalized using RNU44 as an internal control. Data for the qRT‐PCR assays were compared with the results from global expression profiles by the TLDAs system. Data are presented as the mean ± SD of three independent experiments. *P < 0.01 by Student's t‐test. [file MOL2-13-1249-s001.tif]
