# Supplementary material for: New insights into radioresistance in breast cancer identify a dual function of miR‐122 as a tumor suppressor and oncomiR
Source: Mol Oncol. 2019 Apr 18;13(5):1249–67. doi: 10.1002/1878-0261.12483 (PMC6487688; doi:10.1002/1878-0261.12483)
Supplement: Supplementary file 2 — Table S1. Differentially expressed genes in the MCF‐7RR cells transfected with antagomiR‐122. [file MOL2-13-1249-s002.docx]

Supplementary table 1. Differentially expressed genes in the MCF-7RR cells transfected with antagomiR-122

Up-regulated genes

| Number | Gene Symbol | Fold Change | ANOVA *p* value |
| --- | --- | --- | --- |
| 1 | *ADCY10P1* | 2.69 | 0.049564 |
| 2 | *SNORA62* | 2.44 | 0.014568 |
| 3 | *IGLON5* | 1.9 | 0.01885 |
| 4 | *FAM60A* | 1.87 | 0.004694 |
| 5 | *NUP62CL* | 1.64 | 0.045428 |
| 6 | *RNU6-345P* | 1.63 | 0.048881 |
| 7 | *ZNF684* | 1.53 | 0.009666 |
| 8 | *RNA5SP331* | 1.49 | 0.003956 |
| 9 | *ACAA1* | 1.48 | 0.002431 |
| 10 | *KLHL5* | 1.46 | 0.012182 |
| 11 | *RNU2-59P* | 1.43 | 0.023233 |
| 12 | *C20orf203* | 1.43 | 0.028087 |
| 13 | *FBXO48* | 1.39 | 0.03828 |
| 14 | *ZNF304* | 1.37 | 0.009024 |
| 15 | *RNU6-1143P* | 1.37 | 0.033611 |
| 16 | *SPATA41* | 1.36 | 0.038323 |
| 17 | *DOCK11* | 1.35 | 0.004651 |
| 18 | *VIPR2* | 1.35 | 0.027337 |
| 19 | *CATSPER2* | 1.35 | 0.049917 |
| 20 | *SSX8* | 1.34 | 0.000942 |
| 21 | *RBP7* | 1.34 | 0.015545 |
| 22 | *MORC4* | 1.33 | 0.002719 |
| 23 | *CCDC127* | 1.33 | 0.008499 |
| 24 | *KCND3* | 1.33 | 0.026191 |
| 25 | *MIR15A* | 1.33 | 0.041146 |
| 26 | *C21orf128* | 1.32 | 0.043775 |
| 27 | *ZNF611* | 1.31 | 0.037283 |

Down-regulated genes

| Number | Gene Symbol | Fold Change | ANOVA p-value |
| --- | --- | --- | --- |
| 1 | *RNA5SP22* | -2.57 | 0.030472 |
| 2 | *TERF1P2* | -1.72 | 0.037845 |
| 3 | *FLJ45832* | -1.63 | 0.04764 |
| 4 | *SPRR2D* | -1.61 | 0.035229 |
| 5 | *TMEM229A* | -1.59 | 0.015536 |
| 6 | *KLHDC10* | -1.58 | 0.039493 |
| 7 | *MMP26* | -1.57 | 0.028751 |
| 8 | *AKR7L* | -1.56 | 0.033014 |
| 9 | *RNU2-11P* | -1.56 | 0.048154 |
| 10 | *OR7E105P* | -1.55 | 0.01292 |
| 11 | *LCE2C* | -1.55 | 0.01932 |
| 12 | *SNORD116-14* | -1.53 | 0.01283 |
| 13 | *PRH2* | -1.52 | 0.005957 |
| 14 | *FAM207A* | -1.52 | 0.033463 |
| 15 | *LOC613206* | -1.52 | 0.039117 |
| 16 | *RAB40AL* | -1.5 | 0.020366 |
| 17 | *RN7SL799P* | -1.49 | 0.008577 |
| 18 | *SEMA5A* | -1.49 | 0.029573 |
| 19 | *MICAL2* | -1.49 | 0.049496 |
| 20 | *SLC25A18* | -1.47 | 0.001157 |
| 21 | *WFDC13* | -1.47 | 0.003924 |
| 22 | *LOC280665* | -1.47 | 0.008293 |
| 23 | *PODNL1* | -1.46 | 0.027996 |
| 24 | *BHLHA9* | -1.46 | 0.033995 |
| 25 | *RN7SL845P* | -1.45 | 0.008744 |
| 26 | *RNA5SP260* | -1.45 | 0.010754 |
| 27 | *LOC100288160* | -1.44 | 0.000338 |
| 28 | *CXorf65* | -1.44 | 0.025288 |
| 29 | *EMG1* | -1.44 | 0.030918 |

| 30 | *SERP2* | -1.44 | 0.03894 |
| --- | --- | --- | --- |
| 31 | *IGKV6-21* | -1.44 | 0.048681 |
| 32 | *RNA5SP490* | -1.43 | 0.009165 |
| 33 | *MXRA5P1* | -1.43 | 0.016998 |
| 34 | *DUSP8* | -1.43 | 0.023616 |
| 35 | *OR10U1P* | -1.43 | 0.02384 |
| 36 | *RNU6-1244P* | -1.43 | 0.024489 |
| 37 | *RNU6-759P* | -1.42 | 0.001643 |
| 38 | *OR4D10* | -1.42 | 0.002016 |
| 39 | *RN7SL404P* | -1.42 | 0.021392 |
| 40 | *LOC100128035* | -1.42 | 0.025242 |
| 41 | *HTR1E* | -1.42 | 0.026313 |
| 42 | *RNU6-531P* | -1.42 | 0.046175 |
| 43 | *ZNF616* | -1.41 | 0.006054 |
| 44 | *DDR2* | -1.4 | 0.00064 |
| 45 | *IFNL2* | -1.4 | 0.008158 |
| 46 | *RNA5SP43* | -1.4 | 0.008372 |
| 47 | *ACSBG1* | -1.4 | 0.0115 |
| 48 | *OR8B4* | -1.4 | 0.021526 |
| 49 | *LOC100131496* | -1.4 | 0.022992 |
| 50 | *LOC400661* | -1.4 | 0.025557 |
| 51 | *RN7SL708P* | -1.4 | 0.037978 |
| 52 | *TOMM20L* | -1.39 | 0.013789 |
| 53 | *C2CD4A* | -1.39 | 0.027881 |
| 54 | *OR10T1P* | -1.39 | 0.03235 |
| 55 | *KIAA0825* | -1.38 | 0.016549 |
| 56 | *FAM90A27P* | -1.38 | 0.025368 |
| 57 | *C6orf163* | -1.38 | 0.041046 |
| 58 | *ZNF793* | -1.38 | 0.047813 |
| 59 | *MAGEB6P1* | -1.38 | 0.047823 |
| 60 | *DLX4* | -1.37 | 0.001749 |
| 61 | *CD1E* | -1.37 | 0.008049 |
| 62 | *C21orf88* | -1.37 | 0.012189 |
| 63 | *RPL41* | -1.37 | 0.015128 |
| 64 | *FOXD4L6* | -1.37 | 0.015801 |
| 65 | *SNAI1P1* | -1.36 | 0.002469 |
| 66 | *OR2I1P* | -1.36 | 0.004014 |
| 67 | *TFCP2L1* | -1.36 | 0.004789 |
| 68 | *OR1L8* | -1.36 | 0.007069 |
| 69 | *SP7* | -1.36 | 0.009316 |
| 70 | *LIN28A* | -1.36 | 0.047703 |
| 71 | *RNA5SP185* | -1.36 | 0.049989 |
| 72 | *RBPJL* | -1.35 | 0.009438 |
| 73 | *CIDECP* | -1.35 | 0.00976 |
| 74 | *CITED4* | -1.35 | 0.017309 |
| 75 | *LTF* | -1.35 | 0.021755 |
| 76 | *RSPO3* | -1.35 | 0.022451 |
| 77 | *MAP4K1* | -1.35 | 0.023296 |
| 78 | *HRAS* | -1.35 | 0.040605 |
| 79 | *RN7SL307P* | -1.34 | 0.001091 |
| 80 | *TRPM1* | -1.34 | 0.01183 |
| 81 | *IL13* | -1.34 | 0.013097 |
| 82 | *FOXD4L6* | -1.34 | 0.026787 |
| 83 | *FOXD4L6* | -1.34 | 0.026787 |
| 84 | *TNFRSF21* | -1.34 | 0.027477 |
| 85 | *LOC440896* | -1.34 | 0.031084 |
| 86 | *RNU1-94P* | -1.34 | 0.033078 |
| 87 | *KRTAP17-1* | -1.34 | 0.033586 |
| 88 | *SNORD50B* | -1.34 | 0.045892 |
| 89 | *PLA2G10* | -1.34 | 0.047254 |
| 90 | *TTLL13* | -1.33 | 0.00097 |
| 91 | *TMEM194B* | -1.33 | 0.001236 |
| 92 | *PPAPDC3* | -1.33 | 0.012636 |
| 93 | *ZNF18* | -1.33 | 0.016243 |
| 94 | *C17orf47* | -1.33 | 0.02108 |
| 95 | *CAPNS2* | -1.33 | 0.027407 |
| 96 | *PTGDS* | -1.33 | 0.027725 |
| 97 | *IL1R2* | -1.33 | 0.038964 |
| 98 | *RNU6-302P* | -1.33 | 0.040819 |
| 99 | *OR5AP2* | -1.32 | 0.000289 |
| 100 | *VEPH1* | -1.32 | 0.001466 |
| 101 | *DKFZp779M0652* | -1.32 | 0.010157 |
| 102 | *LOC439951* | -1.32 | 0.011456 |
| 103 | *CCL25* | -1.32 | 0.016893 |
| 104 | *SNORD102* | -1.32 | 0.01967 |
| 105 | *SLC10A4* | -1.32 | 0.020593 |
| 106 | *USP20* | -1.32 | 0.0222 |
| 107 | *OR4A13P* | -1.32 | 0.022998 |
| 108 | *TMEM176A* | -1.32 | 0.024448 |
| 109 | *RNU6-561P* | -1.32 | 0.024723 |
| 110 | *ENO3* | -1.31 | 0.002357 |
| 111 | *MB* | -1.31 | 0.002957 |
| 112 | *SLC45A1* | -1.31 | 0.002967 |
| 113 | *RN7SL378P* | -1.31 | 0.005099 |
| 114 | *RIPK1* | -1.31 | 0.005897 |
| 115 | *CNIH3* | -1.31 | 0.006585 |
| 116 | *HEATR3* | -1.31 | 0.008933 |
| 117 | *DTX2* | -1.31 | 0.011503 |
| 118 | *SENP8* | -1.31 | 0.013735 |
| 119 | *ACTA2-AS1* | -1.31 | 0.01574 |
| 120 | *KRT77* | -1.31 | 0.01621 |
| 121 | *C20orf181* | -1.31 | 0.01732 |
| 122 | *EGR4* | -1.31 | 0.018921 |
| 123 | *AREG* | -1.31 | 0.023358 |
| 124 | *ARHGAP23* | -1.31 | 0.024504 |
| 125 | *RN7SL518P* | -1.31 | 0.025984 |
| 126 | *HSPBP1* | -1.31 | 0.030718 |
| 127 | *EPPIN* | -1.31 | 0.032784 |
| 128 | *NUB1* | -1.31 | 0.03411 |
| 129 | *LOC54944* | -1.31 | 0.037601 |
| 130 | *DEAF1* | -1.31 | 0.038737 |
| 131 | *C4B* | -1.31 | 0.03882 |
