# Supplementary material for: New insights into radioresistance in breast cancer identify a dual function of miR‐122 as a tumor suppressor and oncomiR
Source: Mol Oncol. 2019 Apr 18;13(5):1249–67. doi: 10.1002/1878-0261.12483 (PMC6487688; doi:10.1002/1878-0261.12483)
Supplement: Supplementary file 3 — Table S2. Characteristics of the population of breast cancer patients based on expression of the ZNF611 gene. Table S3. Characteristics of the population of breast cancer patients based on expression of the ZNF304 gene. Table S4. Characteristics of the population of breast cancer patients based on expression of the RIPK1 gene. Table S5. Characteristics of the population of breast cancer patients based on expression of the TNFRSF21 gene. Table S6. Characteristics of the population of breast cancer patients based on expression of the DUSP8 gene. Table S7. Characteristics of the population of breast cancer patients based on expression of the HRAS gene. [file MOL2-13-1249-s003.docx]

Supplementary table 2. Characteristics of the population of breast cancer patients based on expression of *ZNF611* gene.

| Characteristics |  | High expression of *ZNF611* | Low expression of *ZNF611* | *P* value |
| --- | --- | --- | --- | --- |
|  | Rank | n=127 (%) | N=129 (%) |  |
| Age | <58 | 67 (52.7) | 57 (44.1) | 0.1701 |
|  | >59 | 60 (47.3) | 72 (55.9) |  |
| Ethnic group | White | 83 (65.3) | 92 (71.3) | 0.6568 |
|  | Black | 9 (7.3) | 7 (5.4) |  |
|  | Asian | 1 (0.8) | 2 (1.5) |  |
|  | American Indian | - | - |  |
|  | NA | 34 (26.7) | 28 (21.7) |  |
| Histological type | Ductal | 98 (77.1) | 103 (79.8) | 0.5040 |
|  | Lobular | 26 (20.4) | 19 (14.7) |  |
|  | Metaplastic | 1 (0.8) | 1 (0.8) |  |
|  | Medullary | 1 (0.8) | 2 (1.5) |  |
|  | Mucinous | 1 (0.8) | 4 (3.1) |  |
| Pathological state | I-II | 75 (59.0) | 58 (44.9) | 0.0240* |
|  | III-IV | 52 (41.0) | 71 (55.1) |  |
| Radiation regimen | Adjuvant | 51 (40.1) | 55 (42.6) | 0.3228 |
|  | Palliative | 1 (0.8) | - |  |
|  | Recurrence | 2 (1.6) | - |  |
|  | NA | 84 (66.1) | 74 (57.4) |  |
| Molecular Classification | Luminal A | 82 (64.5) | 67 (52.0) | 0.2212 |
|  | Luminal B | 23 (18.1) | 26 (20.1) |  |
|  | TNBC | 20 (15,7) | 31 (24.0) |  |
|  | HER2 | - | 1 (0.8) |  |
|  | NA | 2 (1.6) | 4 (3.1) |  |

Supplementary table 3. Characteristics of the population of breast cancer patients based on expression of *ZNF304* gene.

| Characteristics |  | High expression of *ZNF304* | Low expression of *ZNF304* | *P* value |
| --- | --- | --- | --- | --- |
|  | Rank | n=234 (%) | n= 229 (%) |  |
| Age | <58 | 116 (49.6) | 121 (52.8) | 0.5155 |
|  | >59 | 118 (50.4) | 108 (47.2) |  |
| Ethnic group | White | 184 (78.6) | 144 (63.0) | <0.001** |
|  | Black | 15 (6.4) | 66 (29.0) |  |
|  | Asian | 7 (3.0) | 2 (0.87) |  |
|  | American indian | - | 1 (0.43) |  |
|  | NA | 28 (12.0) | 16 (6.7) |  |
| Histological type | Ductal | 184 (79.0) | 167 (73.0) | 0.0538 |
|  | Lobular | 49 (20.5) | 51 (22.2) |  |
|  | Metaplastic | - | 3 (1.3) |  |
|  | Medullary | - | 1 (0.43) |  |
|  | Mucinous | 1 (0.5) | 7 (3.07) |  |
| Pathological state | I-II | 176 (55.5) | 145 (78.0) | 0.0072** |
|  | III-IV | 58 (44.5) | 84 (22.0) |  |
| Radiation regimen | Adjuvant | 37 (45.7) | 28 (34.3) | 0.2378 |
|  | Palliative | - | 1 (1.2) |  |
|  | Recurrence | - | 1 (1.2) |  |
|  | NA | 44 (54.3) | 52 (63.3) |  |
| Molecular Classification | Luminal A | 160 (68.3) | 130 (56.7) | 0.0143* |
|  | Luminal B | 33 (14.1) | 33 (14.4) |  |
|  | TNBC | 25 (10.6) | 49 (21.3) |  |
|  | HER2 | 5 (2.1) | 9 (4.0) |  |
|  | NA | 11 (4.7) | 8 (3.4) |  |

Supplementary table 4. Characteristics of the population of breast cancer patients based on expression of *RIPK1* gene.

| Characteristics |  | High expression of *RIPK1* | Low expression of *RIPK1* | *P* value |
| --- | --- | --- | --- | --- |
|  | Rank | n=90 (%) | n=97 (%) |  |
| Age | <58 | 42 (46.6) | 39 (40.2) | 0.3730 |
|  | >59 | 48 (53.4) | 58 (59.8) |  |
| Ethnic group | White | 54 (60.0) | 62 (64.0) | 0.2217 |
|  | Black | 26 (28.8) | 18 (18.5) |  |
|  | Asian | 1 (1.1) | - |  |
|  | American indian | - | 1 (1.1) |  |
|  | NA | 9 (10.0) | 16 (16.4) |  |
| Histological type | Ductal | 50 (55.5) | 70 (72.2) | 0.1750 |
|  | Lobular | 33 (36.6) | 23 (23.7) |  |
|  | Metaplastic | 1 (1.1) | - |  |
|  | Medullary | 4 (4.4) | 3 (3.0) |  |
|  | Mucinous | 2 (2.2) | 1 (1.1) |  |
| Pathological state | I-II | 49 (54.4) | 51 (52.5) | 0.7981 |
|  | III-IV | 41 (45.6) | 46 (47.5) |  |
| Radiation regimen | Adjuvant | 52 (57.7) | 43 (44.3) | 0.2355 |
|  | Palliative | 1 (1.1) | 1 (1.1) |  |
|  | Recurrence | 2 (2.2) | 1 (1.1) |  |
|  | NA | 35 (38.8) | 52 (53.5) |  |
| Molecular Classification | Luminal A | 63 (70.0) | 55 (56.7) | 0.0526 |
|  | Luminal B | 14 (15.5) | 10 (10.3) |  |
|  | TNBC | 11 (12.2) | 27 (27.8) |  |
|  | HER2 | - | 1 (1.1) |  |
|  | NA | 2 (2.2) | 4 (4.1) |  |

Supplementary table 5. Characteristics of the population of breast cancer patients based on expression of *TNFRSF21* gene.

| Characteristics |  | High expression of *TNFRSF21* | Low expression of *TNFRSF21* | *P* value |
| --- | --- | --- | --- | --- |
|  | Rank | n=246 (%) | n=245 (%) |  |
| Age | <58 | 157 (63.8) | 142 (57.9) | 0.1832 |
|  | >59 | 89 (36.2) | 103 (42.1) |  |
| Ethnic group | White | 179 (72.8) | 153 (62.4) | <0.01** |
|  | Black | 51 (20.7) | 40 (16.3) |  |
|  | Asian | 3 (1.2) | 1 (0.4) |  |
|  | American indian | 2 (0.8) | 5 (2.0) |  |
|  | NA | 11 (4.5) | 46 (18.8) |  |
| Histological type | Ductal | 145 (58.9) | 174 (71.0) | <0.01** |
|  | Lobular | 89 (36.1) | 54 (22.0) |  |
|  | Metaplastic | 4 (1.6) | 10 (4.0) |  |
|  | Medullary | 2 (0.8) | 3 (1.2) |  |
|  | Mucinous | 6 (2.4) | 4 (1.6) |  |
| Pathological state | I-II | 85 (34.5) | 98 (40.0) | 0.2120 |
|  | III-IV | 161 (65.5) | 147 (60.0) |  |
| Radiation regimen | Adjuvant | 197 (80.0) | 201 (82.0) | 0.3953 |
|  | Palliative | 5 (2.0) | 2 (0.8) |  |
|  | Recurrence | 7 (2.8) | 3 (1.2) |  |
|  | NA | 37 (15.0) | 39 (16.0) |  |
| Molecular Classification | Luminal A | 129 (52.4) | 149 (60.8) | <0.01** |
|  | Luminal B | 58 (23.5) | 51 (20.8) |  |
|  | TNBC | 50 (20.3) | 22 (9.0) |  |
|  | HER2 | 2 (0.8) | 4 (1.6) |  |
|  | NA | 7 (2.9) | 21 (8.5) |  |

Supplementary table 6. Characteristics of the population of breast cancer patients based on expression of *DUSP8* gene.

| Characteristics |  | High expression of *DUSP8* | Low expression of *DUSP8* | *P* value |
| --- | --- | --- | --- | --- |
|  | Rank | n=234 (%) | n=229 (%) |  |
| Age | <58 | 110 (47.0) | 91 (39.0) | 0.1145 |
|  | >59 | 124 (53.0) | 138 (61.0) |  |
| Ethnic group | White | 231 (98.8) | 173 (75.5) | <0.01** |
|  | Black | 3 (1.2) | 38 (16.6) |  |
|  | Asian | - | 5 (2.3) |  |
|  | American indian | - | - |  |
|  | NA | - | 13 (5.6) |  |
| Histological type | Ductal | 224 (95.7) | 180 (78.6) | <0.01** |
|  | Lobular | 10 (4.3) | 45 (19.6) |  |
|  | Metaplastic | - | 1 (0.4) |  |
|  | Medullary | - | 2 (0.8) |  |
|  | Mucinous | - | 1 (0.4) |  |
| Pathological state | I-II | 185 (79.0) | 152 (66.3) | <0.01** |
|  | III-IV | 49 (21.0) | 77 (33.7) |  |
| Radiation regimen | Adjuvant | 210 (89.7) | 140 (61.1) | <0.01** |
|  | Palliative | - | 1 (0.4) |  |
|  | Recurrence | - | 1 (0.4) |  |
|  | NA | 24 (10.3) | 87 (37.9) |  |
| Molecular Classification | Luminal A | 12 (5.1) | 152 (66.3) | <0.01** |
|  | Luminal B | 218 (93.1) | 24 (10.4) |  |
|  | TNBC | 2 (0.8) | 42 (18.3) |  |
|  | HER2 | 2 (0.8) | 1 (0.4) |  |
|  | NA | - | 10 (4.3) |  |

Supplementary table 7. Characteristics of the population of breast cancer patients based on expression of *HRAS* gene.

| Characteristics |  | High expression of *HRAS* | Low expression of *HRAS* | *P* value |
| --- | --- | --- | --- | --- |
|  | Rank | n=99 (%) | n=96 (%) |  |
| Age | <58 | 34 (34.3) | 43 (44.7) | 0.1356 |
|  | >59 | 65 (65.7) | 53 (55.3) |  |
| Ethnic group | White | 58 (58.6) | 49 (51.0) | 0.4011 |
|  | Black | 20 (20.2) | 31 (32.3) |  |
|  | Asian | 5 (5.0) | 4 (4.1) |  |
|  | American indian | 1 (1.0) | - |  |
|  | NA | 15 (15.2) | 12 (12.5) |  |
| Histological type | Ductal | 72 (72.7) | 69 (71.8) | 0.3567 |
|  | Lobular | 24 (24.2) | 20 (20.8) |  |
|  | Metaplastic | 1 (1.0) | 2 (2.0) |  |
|  | Medullary | 1 (1.0) | - |  |
|  | Mucinous | 1 (1.0) | 5 (5.3) |  |
| Pathological state | I-II | 67 (67.6) | 63 (65.6) | 0.7612 |
|  | III-IV | 32 (32.4) | 33 (34.4) |  |
| Radiation regimen | Adjuvant | 50 (50.5) | 28 (29.1) | <0.01** |
|  | Palliative | - | - |  |
|  | Recurrence | 1 (1.0) | - |  |
|  | NA | 48 (48.5) | 68 (70.9) |  |
| Molecular Classification | Luminal A | 57 (57.5) | 61 (63.5) | 0.2646 |
|  | Luminal B | 17 (17.1) | 12 (12.5) |  |
|  | TNBC | 23 (23.2) | 18 (18.7) |  |
|  | HER2 | 1 (1.0) | - |  |
|  | NA | 1 (1.0) | 5 (5.2) |  |
